# Discrepancies in the Tumor Microenvironment of Spontaneous and Orthotopic Murine Models of Pancreatic Cancer Uncover a New Immunostimulatory Phenotype for B Cells

**DOI:** 10.3389/fimmu.2019.00542

**Published:** 2019-03-27

**Authors:** Sarah Spear, Juliana B. Candido, Jacqueline R. McDermott, Cristina Ghirelli, Eleni Maniati, Stephen A. Beers, Frances R. Balkwill, Hemant M. Kocher, Melania Capasso

**Affiliations:** ^1^Centre for Cancer and Inflammation, Barts Cancer Institute, Queen Mary University of London, London, United Kingdom; ^2^Department of Pathology, University College London Hospital, London, United Kingdom; ^3^Antibody and Vaccine Group, Centre for Cancer Immunology, University of Southampton Faculty of Medicine, Southampton, United Kingdom; ^4^Centre for Tumor Biology, Barts Cancer Institute, Queen Mary University of London, London, United Kingdom; ^5^German Center for Neurodegenerative Diseases (DZNE), Bonn, Germany

**Keywords:** B cells, immunoglobulins, tumor microenvironment, pancreatic cancer, murine models

## Abstract

B cells are salient features of pancreatic ductal adenocarcinoma (PDAC) tumors, yet their role in this disease remains controversial. Murine studies have indicated a protumoral role for B cells, whereas clinical data show tumor-infiltrating B cells are a positive prognostic factor, both in PDAC and other cancers. This disparity needs to be clarified in order to develop effective immunotherapies. In this study, we provide new evidence that reconcile human and mouse data and highlight the importance of using relevant preclinical tumor models when assessing B cell function. We compared B cell infiltration and activation in both a genetic model of murine PDAC (KPC mouse) and an injectable orthotopic model. A pronounced B cell infiltrate was only observed in KPC tumors and correlated with T cell infiltration, mirroring human disease. In contrast, orthotopic tumors exhibited a relative paucity of B cells. Accordingly, KPC-derived B cells displayed markers of B cell activation (germinal center entry, B cell memory, and plasma cell differentiation) accompanied by significant intratumoral immunoglobulin deposition, a feature markedly weaker in orthotopic tumors. Tumor immunoglobulins, however, did not appear to form immune complexes. Furthermore, in contrast to the current paradigm that tumor B cells are immunosuppressive, when assessed as a bulk population, intratumoral B cells upregulated several proinflammatory and immunostimulatory genes, a distinctly different phenotype to that of splenic-derived B cells; further highlighting the importance of studying tumor-infiltrating B cells over B cells from secondary lymphoid organs. In agreement with the current literature, genetic deletion of B cells (μMT mice) resulted in reduced orthotopic tumor growth, however, this was not recapitulated by treatment with B-cell-depleting anti-CD20 antibody and, more importantly, was not observed in anti-CD20-treated KPC mice. This suggests the result from B cell deficient mice might be caused by their altered immune system, rather than lack of B cells. Therefore, our data indicate B cells do not favor tumor progression. In conclusion, our analysis of relevant preclinical models shows B cells to be active members of the tumor microenvironment, producing immunostimulatory factors that might support the adaptive antitumor immune response, as suggested by human PDAC studies.

## Introduction

Following a first observation in experimental fibrosarcomas in 1978, a tumor-promoting role for B cells has been demonstrated in multiple murine models of cancer (1–6). There are, however, contrary murine studies which showed that B cells infiltrate tumors and positively influence T cell proliferation and cytokine production *in situ* (7–9), thus demonstrating antitumoral activity.

From the literature, it appears the role of B cells may be highly dependent on the tumor type and location, the mode of B cell depletion and whether the analysis was performed on peripheral or tumor-infiltrating B cells. There is also clear disparity between murine and clinical data, where many human cancers are heavily infiltrated by B cells and this correlates with improved prognosis, for example in breast cancer (10, 11), ovarian (12), non-small cell lung cancer (13), esophageal and gastric adenocarcinoma (14), colorectal cancer (15). The antitumoral function of B cells in human cancers is largely attributed to their ability to differentiate into plasma cells (16), produce antitumoral immunoglobulins (17, 18) and interact with T cells in tertiary lymphoid structures (TLS) (13, 19, 20). Therefore, a full understanding for the role of B cells in tumor development is still lacking.

In this study we focus on pancreatic ductal adenocarcinoma (PDAC), a cancer with a dismal prognosis of <4% survival over 5-years, which has not significantly changed over the last 50 years (21). As only 20% of PDAC patients are eligible for surgical resection (22), access to samples is limited and thus research heavily relies on murine models. PDAC research utilizes different types of immunologically intact preclinical murine models, the gold-standard of which is the KPC mouse (*Kras*^*G*12*D*/+^; *Trp53*^*R*172*H*/+^; *Pdx-1-Cre*), which spontaneously develops PDAC over time and faithfully recapitulates human disease in terms of progression through PanINs (Pancreatic Intraepithelial Neoplasia), desmoplastic stromal reaction and metastatic sites with a median survival of 5 months (23). Another related model, the KC mouse, expresses only mutant *Kras* and consequently develops PDAC at a significantly slower rate, where the majority of mice under 5 months are histologically normal (24). A faster and more readily accessible model is the orthotopic model, which is generated by the injection of syngeneic tumor cells into the healthy pancreas. Orthotopic tumors form rapidly in approximately 1 month (25) and as such, contain less stroma and instead are more tumor-cell rich, most probably owing to the accelerated disease progression (25, 26).

Recent data obtained with orthotopic and KC mice has demonstrated B cells were tumor-promoting, using B cell-deficient mice (27, 28). A subset of IL-35-secreting CD1d^hi^CD5^+^ B cells was shown to arise in orthotopic and KC PDAC and orthotopic tumor growth in B cell-deficient mice was reduced (28). Additionally, splenic-derived B cells isolated from tumor-bearing mice polarized macrophages to a Th2 phenotype and a Bruton's tyrosine kinase (BTK) inhibitor, which targets B cells and myeloid cells, demonstrated reduced tumor growth in the orthotopic model (27). An additional study found hypoxia-inducing factor (HIF) 1α-deficient KC mice had enhanced PanIN progression at early stages, which could be curtailed when B cell-depletion was commenced in the early phase of life, prior to tumor appearance (29). Yet, as for other cancers, this is discordant with data from human PDAC, where a high B cell infiltrate is associated with better prognosis (20, 30), especially the presence of B cells clustered in Tertiary Lymphoid Structures (TLS) (20). Furthermore, autoantibodies have been identified in PDAC patient serum (31, 32), PDAC-derived IgE has shown ADCC activity *in vitro* (33) and anti-MUC1 autoantibodies correlated with improved prognosis in pancreatic cancer patients (34).

Considering their potential impact on patient prognosis, it is critical to better understand the role of B cells in PDAC in order to further refine anti-tumor immunotherapies. In this study, we aimed to compare the role of B cells from different mouse models of PDAC, starting from the most relevant preclinical model, the KPC mouse. We show here that the KPC model indeed closely reflects human PDAC and is strongly infiltrated by B cells, presenting signs of germinal center entry, B cell memory and plasma cell expansion, immunoglobulin production, and deposition. Crucially, B cell infiltration and immunoglobulin deposition occur to a far lesser extent in orthotopics. Furthermore, we show for the first time how the collective phenotype of tumor-infiltrating B cells is actually proinflammatory, rather than immunosuppressive, and furthermore is distinctly different to that of splenic-derived B cells. In agreement with previous studies, genetic B cell deletion reduces orthotopic tumor growth, however, we additionally show that B cell depletion via anti-CD20 treatment does not. More importantly, B cell depletion in the KPC model also does not impede tumor growth, indicating that, overall, B cells do not favor tumor progression. Taken together, our results indicate the importance to use relevant mouse models to study the tumor microenvironment of PDAC. Furthermore, they show for the first time that tumor-infiltrating B cells have a proinflammatory phenotype, which might support the overall immune response against the tumor, as human PDAC studies have also indicated.

## Materials and Methods

### Animal Experiments

Animal procedures were carried out in accordance with the U.K. Home Office Animal and Scientific Procedures Act 1986 and the European Directive 2010/63/EU, reviewed and approved by the UK Home Office (project licenses 70/7411, 70/7449 and PBE3719B3). Male and female μMT mice (C57BL/6 background) were generated as described (35). Male and female KPC mice (23) were generated in house by crossing *Lox-STOP-Lox* (LSL) *Kras*^*G*12*D*/+^ and *LSL-Trp53*^*R*172*H*/+^ (C57BL/6/129/SVJae) with *Pdx-1-Cre* (C57BL/6) mice. Control mice were aged-matched healthy *Pdx-1-Cre*. For orthotopic experiments using anti-CD20, 10–12-week-old female C57BL/6 mice (Charles River, U.K.) were used.

### Orthotopic Injection of Tumor Cells

The KPC-derived PDAC cell line (TB32048) previously generated from a female C57BL/6 KPC mouse was provided as a generous gift from David Tuveson laboratory. TB32048 was cultured for 3–4 passages at maximum 80% confluency in 10% fetal calf-serum (#A15-104, GE Healthcare) in DMEM (#E15-810, PAA) + 100 μg/ml penicillin/streptomycin (#P11-G10, PAA) in a T175 flask in standard conditions (37°C, 5% CO_2_) and tested regularly in house for mycoplasma (#rep-pt1, Invivogen and #LT07-710, Lonza). Cells were dissociated using 0.1% trypsin (PBS) (#594-18C, Sigma) for 10 min at 37°C and resuspended in PBS and BD Matrigel™ Basement Membrane Matrix High Concentration (#354248, BD Biosciences) in a ratio of 1:1. 1,000 cells in 5 μl was injected into the pancreas using a Hamilton® syringe, 700 series (#10100332, Fisher Scientific). For the experiments used to assess CD138^hi^ cells and anti-CD20 at day 10, 10,000 cells in 30 μl Matrigel, or Matrigel only sham surgery was performed. The peritoneal wall was sutured using 6/0-gauge coated vicryl sutures™ (#W9500T, Ethicon) and skin closed using two 9 mm Clay Adams Clips (#IN015A, VetTech Solutions) and an Autoclip® applier (#IN015B, VetTech Solutions).

### Depletion of B Cells Using an Anti-CD20 Antibody

Mice with orthotopic tumors were treated with either a B-cell-depleting anti-CD20 (mouse IgG2a, clone 18B12) or IgG2a isotype control (clone WR17), produced in house (36). Treatments were administered on day−8,−2, 14, with orthotopic surgery performed on day 0. Mice were culled on days 26–27. KPC mice were treated with either 250 μg of anti-CD20 antibody (mouse IgG2a) to deplete B cells or with IgG2a isotype control (anti-ragweed 1428). Both antibodies were provided as a gift from Genentech, however, for the last batch of experiments, the Genentech isotype control was not sufficient and a mouse IgG2a from BioXcell was used instead (clone C1.18.4, lot 5518/1214) (#BE0085, BioXcell). KPC mice were palpated at least once per week for a pancreatic tumor from approximately 70 days old. When a tumor was detected, treatment with either PBS, anti-CD20 or mouse IgG2a commenced between 1 and 7 days later. Treatments were given in 200 μl sterile PBS once per week for 5 (*n* = 20) or 6 weeks (*n* = 8) and mice were culled a week later, at which point most had developed small tumors (*n* = 20). The Genentech antibodies were also used for the orthotopic experiment, where anti-CD20 or IgG was administered at day 10 and mice culled at day 23 (endpoint).

### Processing of Organs for Flow Cytometry

Organs were passed through a 70-μm-pore size cell strainer (#11597522, Fisher). Red blood cell lysis was performed for blood and spleen samples using RBC Lyse buffer (#555899, BD Biosciences) for 10 min at RT. Tumors were digested in 5 ml 2.0 mg/ml collagenase (#C9263, Sigma) (DMEM), 0.025 mg/ml DNase (#D4513, Sigma) at 37°C under agitation for 20 min and passed through a 70 μm cell strainer to achieve a single cell suspension. 0.5–2.0 × 10^6^ cells were incubated with anti-CD16/32 FcR Block (1:200) (#553142, BD Biosciences) for 15 min followed by 50 μl containing labeled antibodies (Supplementary Table S1) for 30 min, all at 4°C. Cells were washed in FACS buffer (5% BSA, 2mM EDTA, PBS) and incubated with fixable viability dye (FVD506) at 1:200 in PBS (#65-0866, eBioscience) at 4°C for 20 min. After further washing in FACS buffer, they were incubated for 20 min in 100 μl of 2% paraformaldehyde/FACS buffer (#BX1143CB0201, Adams) at 4°C. Cells were washed and analyzed on BD LSR Fortessa™. For qPCR B cells were sorted from spleens and tumors as DAPI^−^ CD45^+^ CD19^+^ (with the exclusion of CD138^+^ in the spleens) using fluorescence-activated cell sorting. Cells were stained as above but approximately 100 μl of FcR block and 100 μl 2X antibody master mix was used per 10 × 10^6^ cells. The viability dye DAPI (#D9542, Sigma) was added at 2 μg/ml immediately before sample acquisition on the BD FACS Aria II and samples were collected in 1 ml sterile FBS before RNA extraction.

### Immunofluorescence Staining of Mouse Sections

All immunofluorescence (IF) was carried out at RT. 4–7 μm sections were prepared from frozen samples, which were thawed then fixed with 4% paraformaldehyde solution (#BX1143CB0201, Adams) for 20 min at RT. Sections were washed 3x in 0.1% PBS-Tween 20 (PBS-T) and permeabilized in 0.1% TritonX100 solution for 5 min. Then they were washed and blocked using a blocking buffer of 5% goat serum, 2.5% BSA in PBS for 1 hr. B220, E-Cadherin, EpCAM, IgG1, IgG2a/b, IgG3, and IgM were added in blocking buffer for 1 hr (details in Supplementary Table S2 and isotypes in Supplementary Table S3) then washed 3x in PBS-T, 1x PBS, 1x H_2_O for 5 min. Sections were stained with DAPI (1:10,000 5 min in H_2_0) and mounted with Prolong Gold Antifade with DAPI (# P36931, Invitrogen) and stored at 4°C. Images were acquired on LASER scanning microscopes 510 and 710 (Zeiss) and processed using Image J. The colocalization coefficient was generated on the raw LSM files on Zen 2009 software.

### Immunohistochemistry Staining of Mouse Sections

Immunohistochemistry (IHC) was carried out at RT. Frozen sections were thawed and fixed for 20 min in 4% paraformaldehyde solution, then permeabilized in 0.1% TritonX100 solution for 5 min and washed once in 0.1% PBS-Tween (PBS-T). One hundred μl of Dako dual endogenous enzyme block (#S2003, Dako) was added for 15 min at RT and washed 1x in PBS-T, followed by a blocking step in 100 μl 5% goat serum, 2% BSA in PBS (blocking buffer) for 1 hr. The primary antibody (B220, CK8) in blocking buffer was added for 1 hr (details in Supplementary Table S2 and isotypes in Supplementary Table S3). Slides were washed 3x in PBS-T, then secondary anti-rat or anti-rabbit antibodies (1:200) conjugated to biotin in 100 μl blocking buffer were incubated for 1 hr (Supplementary Table S4). Slides were washed 3x in PBS-T and 1x in PBS. Endogenous peroxidase activity was blocked by a 20 min incubation in 250 ml 99.9% Methanol (#M/4000/PB17, Fisher Scientific) and 5 ml 30% H_2_O_2_ (#H/1800/15, Fisher Scientific). Slides were then washed 3x in PBS-T. The VECTASTAIN Elite ABC kit (standard) (#PK-6100, Vector Labs), was prepared as per manufacturer's instructions. Briefly 2 drops of solution “A” and 2 drops of solution “B” were added to 5 ml of PBS, vortexed and stored on ice prior to using, then incubated for 30 min and washed 2x in PBS-T and 1x in PBS. SIGMAFAST™ 3,3'-Diaminobenzidine (DAB) tablets (#D4293, Sigma) were prepared as per manufacturer's instructions. Briefly one DAB tablet and one Tris/peroxide tablet was added to 5 ml doubled distilled H_2_0 and filtered (0.45 μm) before use. DAB was applied to sections for approximately 4–10 min). Slides were washed in double distilled H_2_O. Hematoxylin (#1.05175.2500, VWR) was applied for 1 min then washed until clear in distilled water. Tap water was used to blue tissue for 30 s−1 min. Slides were then dehydrated by 3 min in 95% ethanol, 2x 3 min in 100% ethanol and 5 min in xylene. Coverslips were mounted using DPX solution (#06522, Sigma). B220 and CK8 were quantified using Definiens “Tissue Studio” 64 software.

### RNA Extraction, cDNA Synthesis, and qPCR

Sorted B cells were vortexed for 1 min in 350 μl of RLT buffer per 3 × 10^6^ cells and stored at −80°C. RNA extraction was performed using Qiagen RNeasy micro kit (#74004, Qiagen) as per manufacturer's instructions. RNA concentration and integrity were measured using the Nanodrop and the integrity and concentration of the RNA was in some cases further confirmed using an Agilent RNA 6000 Pico Kit (#5067-1513, Agilent), according to manufacturer's instructions. For experiments using the Qiagen RT2 profiler, cDNA synthesis was performed using the RT2 First Strand Kit (#330401, Qiagen) as per manufacturer's instructions and qPCR performed using the RT^2^ Profiler™ PCR Array Mouse Cancer Inflammation & Immunity Crosstalk (#PAMM-181Z-E-4, Qiagen) performed on the C1000 Touch Thermal Cycler (#CFX384, Bio- Rad), using cycling conditions recommended by the manufacturer. All additional qPCR was performed using reagents from Applied Biosystems. For additional qPCR, RNA was extracted as before. cDNA was generated using the High Capacity reverse transcription kit (#4368814, Applied Biosystems) as per manufacturer instructions. qPCR reaction was performed using a master mix of 9 μl cDNA, 10 μl iTaq (#1725134, Bio-Rad) and 1 μl primer (Applied Biosystems). The primers used were *Cxcl2* Mm00436450_m1, *Ebi3* Mm00469294_m1, *Gapdh* Mm99999915_g1, *Il10* Mm00439614_m1, *Il12a* Mm00434169_m1, *Il12b* Mm00478374_m1, and *Ptgs2* Mm00478374_m1. The reaction was performed on a Step One Plus Thermal Cycler (Applied Biosystems) using the following cycling conditions: 2 min 50°C x1, 10 min 95°C x1, 15 s 95°C x40 cycles, 1 min 60°C. The raw Ct values were exported for subsequent analysis. The ΔCt was calculated by subtracting the Ct of the housekeeping genes *Gapdh* from each Ct value i.e., ΔCt = Ct *Gene* – Ct *Gapdh* and then expressed as 2^∧^(−ΔCt). For data generated using the RT2 Profiler array, a heatmap was generated in the statistical programming language R (version 3.1.3), using the gplots package. It was constructed using row z-scores of log2 transformed gene expression values. Color-coding corresponds to row z-scores, columns represent individual samples and a row dendrogram was included to illustrate clustering relationships of gene expression.

### Immunoglobulin Concentration in Plasma and Pancreas

Plasma and pancreas or pancreatic tumor were harvested from healthy *Pdx-1-Cre* mice and KPC mice at endpoint. Protein lysates of pancreas and tumor tissue were prepared by cutting and weighing the tissue on dry ice, then lysing in ice-cold lysis buffer: 150 mM NaCl, 20 mM Tris pH7.5, 1 mM EDTA, 1 mM EGTA, 1% Triton X-100 with 2 protease inhibitor tablets (#1836170, Roche), 200 μl phosphatase inhibitor II (#P-5726, Sigma) and 200 μl phosphatase inhibitor III (#P-0044, Sigma) per 10 ml of lysis buffer. Lysis buffer was added 1 ml per 75 mg tissue and digested in M-tubes (#130-093-236, Miltenyi) in a gentleMACS™ Dissociator on protein_01 setting (#130-093-235, Miltenyi). Samples were centrifuged at 244 rcf for 2 min and stored on ice, then sonicated in 10 s bursts at 40% amplitude whilst on ice. Samples were then rotated at 4°C for 30 min and centrifuged at 16.1 rcf for 15 min at 4°C. The final supernatant was aliquoted and stored at −80°C and the pellet discarded. The concentration of immunoglobulins IgA, IgG1, IgG2a, IgG2b, IgG3, and IgM was detected using the Mouse Isotyping Panel 1 kit (#K15183B-1, Mesoscale Discovery (MSD), as per manufacturer's instructions and analyzed on an MSD Model S1250 2400. The concentration of IgE was determined using the Mouse IgE ELISA MAX Standard (#432401, Biolegend). For the MSD plate, plasma samples were diluted at 1:5,000 and protein lysates were diluted 1:200 in 1% BSA/PBS. For the IgE ELISA, the plasma and protein lysates were diluted at 1:100 in 1% BSA/PBS.

### Detection of Antibody Immune Complexes in Serum and Pancreas of KPC Mice

The concentration of C1q-Ig immune complexes was analyzed using the Mouse CIC Ig's (total A+G+M) ELISA kit (#5900, Alpha Diagnostic International). Serum was prepared by taking blood from healthy and KPC mice at endpoint. Blood was allowed to clot for 1 hr at RT followed by centrifugation at 16.1 rcf for 10 min, serum was removed and stored at −80°C. Protein lysates of pancreas tissue were made as described above (see immunoglobulin concentration in plasma and pancreas). Samples were thawed on ice and diluted 1:100 in H_2_O, 100 μl were used for the ELISA assay (in duplicate), which was performed as per manufacturer's instructions. Optical density (OD) was measured at 450 nm.

### Stimulation of T Cells and B Cells *ex vivo*

In order to detect intracellular cytokines IFNɤ and TNFα and proliferation (Ki67) in CD4^+^ and CD8^+^ T cells (antibody details in Supplementary Table S1), 2 × 10^6^ cells from digested orthotopic tumors were cultured in a 96-well U-bottomed plate for 5 hr with Cell Stimulation Cocktail (#00-4970-03, eBioscience), with a final concentration of 0.081 μM phorbol 12-myristate 13-acetate (PMA) and 1.34 μM ionomycin (#00-4980-03, eBioscience). After 1 hr incubation, cell transport inhibitor cocktail was added, at a final concentration of 1.06 μM Brefeldin and 2.0 μM Monensin (#00-4980-03, eBioscience) for the remaining 4 hr incubation. After 5 hr, the cells were transferred to a v-bottomed plate on ice and stained for flow cytometry. To detect cytoplasmic IL-10 in B cells, cells from digested pancreatic tumors were plated in 10 μg/ml LPS (#L2630, Sigma), PMA and Brefeldin for 5–12 hr, at which point they were stained for flow cytometry.

### Flow-Cytometry Intracellular (Cytoplasmic) Staining

Cells were prepared for flow cytometry as previously described above. The intracellular fixation and permeabilization buffer kit (#88-8824, eBioscience) was used for intracellular (cytoplasmic) staining. After extracellular staining and viability dye stain had been performed, 100 μl of intracellular (IC) fixation buffer were added for 1 hr at RT. Following this, the IC buffer was washed twice with permeabilization buffer. The intracellular antibody was diluted in permeabilization buffer for 20–60 min. Cell pellets were resuspended in 100 μl FACS buffer and stored at 4°C until acquisition on the flow cytometer, performed on the same or the following day.

### Intranuclear Staining for Flow Cytometry

Cells were prepared for flow cytometry as previously described. For intranuclear staining of markers such as FOXP3 and Ki67 (antibody details in Supplementary Table S1), the Foxp3/transcription factor fixation/permeabilization concentrate and diluent kit (#00-5523, eBioscience) were used. After extracellular staining and viability dye stain had been performed, 100 μl of fixation/permeabilization buffer was added for 1 hr at RT, then washed twice with 100 μl of permeabilization buffer. The intranuclear antibody mix was diluted in permeabilization buffer and incubated for 60 min. The cells were then washed with 100 μl permeabilization buffer and with 100 μl FACS buffer. Cell pellets were resuspended in 100 μl FACS buffer and stored at 4°C until acquisition on the flow cytometer, performed on the same or the following day.

### Pathology Analysis of Tumors

The percentage of PDAC in the tissue of KPC tumors post anti-CD20 treatment was analyzed blind by a Consultant Histopathologist, assessing whole sections (Dr. Jacqueline R. McDermott, Department of Pathology, University College London Hospital). Similarly, PanIN lesions were quantified by analyzing 10 high-powered fields at 20X magnification, blind.

### Data Presentation and Statistical Analysis

Immunofluorescence images were analyzed using Image J (Java) and bright field images were analyzed using Panoramic Viewer (3DHISTECH) software and Zen 2 (Carl Zeiss). Graphic representation of data and statistical analysis was performed using GraphPad Prism Version 5. Data was tested for normality using the Kolmogorov-Smirnov test. If the data were normally distributed, then an unpaired *t*-test or One-Way ANOVA was used with Bonferroni's post-test. Non-parametric data were tested using a Mann-Whitney test or Kruskal-Wallis and Dunn's post-test. A cut off *p* < 0.05 was used to define significance.

Additional tables detailing the antibody clones and respective product codes are available in Supplementary Material.

## Results

### B Cells Display Marked Infiltration and Immunoglobulin Deposition in Murine KPC PDAC, Not Seen in Orthotopic Tumors

**Figure 1:**
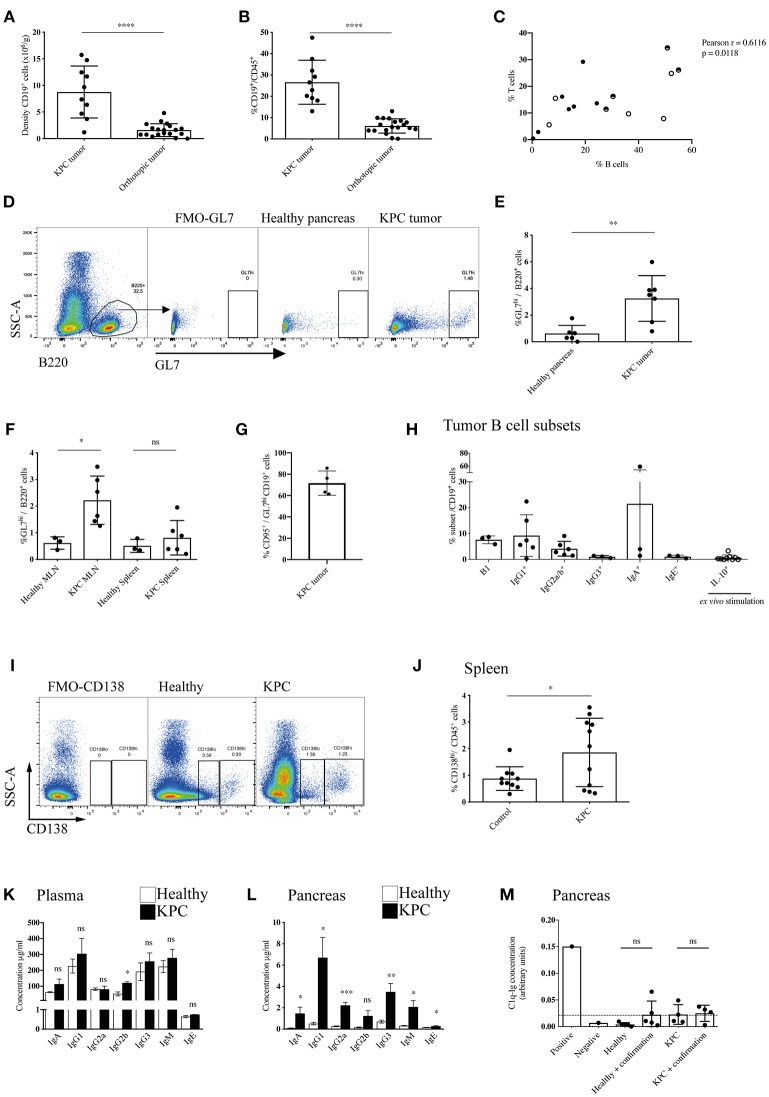
Enhanced recruitment and maturation of B cells in KPC compared to orthotopic tumors. **(A)** Density (number of cells per gram tissue) of CD45^+^ CD19^+^ B cells quantified by flow cytometry in KPC tumors (*n* = 10) and orthotopic tumors (*n* = 19). **(B)** Proportion of CD19^+^ B cells out of CD45^+^ cells quantified by flow cytometry in KPC tumors (*n* = 10) and orthotopic tumors (*n* = 19). **(C)** Proportion of B cells (CD19^+^/B220^+^) and T cells (CD3^+^) out of CD45^+^ cells in KPC mice. Black circles: untreated KPC mice; Half circles: PBS-treated KPC mice at endpoint; white circles: PBS-treated KPC mice not at endpoint. The association was assessed using a Pearson correlation. **(D)** Flow cytometry gating strategy for activated B cells by GL7^hi^ expression on CD45^+^ B220^+^ B cells, with fluorescence minus one (FMO) control used to assess background. **(E,F)** Flow cytometry quantification of proportion of activated B cells by GL7^hi^ cells out of total B220^+^ B cells in **(E)** the healthy pancreas (*n* = 6) and KPC tumor (*n* = 7) and **(F)** in the MLN and spleen or healthy (*n* = 3) and KPC (*n* = 6) mice. Mean + standard deviation (SD), Unpaired *t*-test. **(G)** Flow cytometry quantification of the percentage of CD95^+^ cells out of GL7^hi^ CD19^+^ cells in KPC tumors (*n* = 4). **(H)** Flow cytometry quantification of B cell subsets out of total B cells isolated from KPC tumors: B1 cells (CD11b^+^ B220^+^) (*n* = 3), memory IgG1 (*n* = 6), memory IgG2a/b (*n* = 6), memory IgG3 (*n* = 3), memory IgA (*n* = 3), memory IgE (*n* = 3), IL-10^+^ B cells, post *ex vivo* stimulation with LPS, and PMA (*n* = 9). **(I)** Flow cytometry gating strategy for plasmablasts CD138^lo^ and plasma cells by CD138^hi^ expression on CD45^+^ cells, with FMO control used to assess background. **(J)** Flow cytometry quantification of proportion of CD138^hi^ plasma cells out of total CD45^+^ immune cells in the spleen of healthy (*n* = 10) and KPC (*n* = 11) mice. **(K,L)** Concentration of immunoglobulin isotypes analyzed by Mesoscale (IgA - IgM) and ELISA (IgE) present in **(K)** the plasma and **(L)** pancreas tissue lysates of healthy (*n* = 5) (white) and KPC (*n* = 5) mice (black). **(M)** Relative concentration of C1q-Ig immune complexes as measured by ELISA. Negative and positive controls were provided by the ELISA kit manufacturer, dashed line represents threshold for positivity. The concentration of immune complexes was measured in healthy control pancreata (*n* = 5) and KPC tumors (*n* = 4). A confirmation test that disrupts ICs was performed for each sample as recommended by the manufacturer, however, results show no difference since no ICs were detected. Each data point represents an individual mouse sample, mean and SD are also indicated. Statistical significance was analyzed by unpaired *t*-test where ^*^p < 0.05, ^**^p < 0.01, ^***^p < 0.001 and ^****^p < 0.0001.

In order to dissect the discrepancy regarding the role of B cells in human and murine models of PDAC, we started by assessing the total B cell infiltration in KPC and orthotopic tumors (Figures 1A,B and Supplementary Figure S1A). We observed a substantial infiltration of B cells into KPC tumors, which was significantly higher (6-fold) than that seen in orthotopic tumors at endpoint, both in density (Figure 1A) and proportion of immune infiltrate (Figure 1B). The B cell proportion observed in KPC tumors was comparable to that reported in PDAC patients (27). B cells largely organized in clusters in the stroma of KPC tumors (Supplementary Figure S1B), whereas orthotopic tumors were poorly infiltrated (Supplementary Figure S1C). Furthermore, B cell infiltration significantly correlated with T cell infiltration in KPC tumors (Figure 1C) but not in orthotopic (Supplementary Figure S1D), which showed lower infiltration of both B and T cells. We wondered if the substantial infiltration in KPC tumors was a result of antigen encounter. Following antigen encounter, B cells become activated and can enter germinal centers, leading to B cell proliferation and differentiation to either memory cells or plasma cells producing high-affinity immunoglobulin clones (37, 38). In line with this, we found a significant increase in the GL7^hi^ B cell frequency, a marker of activated B cells (Figure 1D), in both the tumor (Figure 1E) and the tumor-draining mesenteric lymph nodes of KPC mice (Figure 1F) when compared to healthy murine pancreas or lymph nodes, suggesting an active B cell response within the tumor in this model. However, we did not see a significant increase in GL7^hi^ B cells in the spleen (Figure 1F). Most GL7^hi^ B cells were positive for CD95 (Figure 1G and Supplementary Figure S1E), indicating they were part of a germinal center. This was further confirmed in additional samples with the alternative gating strategy of CD95^+^ CD38^−^ staining in KPC tumors (Supplementary Figure S1F). Other subsets such as B1 cells (CD11b^+^) (mean 7.6% ± SEM 0.9) and IgG1^+^(9.2% ± 3.3), IgG2a/b^+^(4.1% ± 1.1) and IgA^+^ (21.5% ±18.8) memory B cells were also present, whereas IgG3^+^(1.0% ± 0.3) and IgE^+^ (1.1% ± 0.3) memory B cells and IL-10^+^ (0.7% ± 0.4) regulatory B cells were present in very low numbers in KPC tumors (Figure 1H and Supplementary Figures 1G–M). Plasma cells were only present in KPC tumors at a very low density, not significantly different from healthy pancreas, which normally lacks plasma cells (Supplementary Figure S1N), whereas they were significantly increased in the spleen (Figures 1I,J). However, plasmablasts were not significantly upregulated in the spleen (Supplementary Figure S1O). In contrast, in a small cohort of orthotopic mice, no expansion of splenic plasma cells was observed, when compared to aged-matched healthy and sham surgery controls (Supplementary Figure S1P).

Next, we wanted to assess if the plasma cells were producing immunoglobulins (Igs) and if these were reaching the tumor, therefore we analyzed all subclasses in the blood and tumor lysates of KPC mice. We found a slight increase of IgGs in KPC mice blood, which reached statistical significance only for IgG2b (Figure 1K). Crucially, we found a significant increase in all but one subclass (IgG2b) in KPC tumors compared to healthy pancreas (Figure 1L). The range of immunoglobulin subclasses produced indicated a broad-range of cytokine and antigen exposure. Interestingly, IgG1 is the most dominant subclass found in the blood (Figure 1K) and was similarly most strongly increased in the tumor; suggesting a Th2-cytokine dominance may be present during B-cell class switching, which has been described for PDAC (39). Additionally, we found a significant increase in tumor IgG2a (Figure 1L), regarded as the most pro-inflammatory subclass, providing the most effective antiviral or pathogenic response (40, 41). Although at a lower concentration than the other subclasses, IgE was also significantly increased within the tumor (Figure 1L), confirming data from a small cohort of human PDAC patients, where patient-derived IgE was found upregulated and exerted antitumoral activity via ADCC (33). Immunoglobulins target their bound antigen and this can lead to their degradation/elimination. One of the possible mechanisms by which they do this is through the formation of immune complexes (IC). Classically, during an infection, IC lead to activation of the complement cascade and the lysis of a target cell, or they target the antigen for phagocytosis (42). In cancer, however, IC have been shown to be tumor-promoting, by stimulating macrophage IL-10 secretion and potentiating an immunosuppressive microenvironment (43, 44). In order to assess if IgG were bound in immune complexes, thus contributing to an immunosuppressive environment in KPC mice, we analyzed IC content in the serum and tumors with a C1q-IgG ELISA, as previously reported (45). However, we found no evidence of increased IC formation at either site (Figure 1M and Supplementary Figure S1Q).

**Figure 2:**
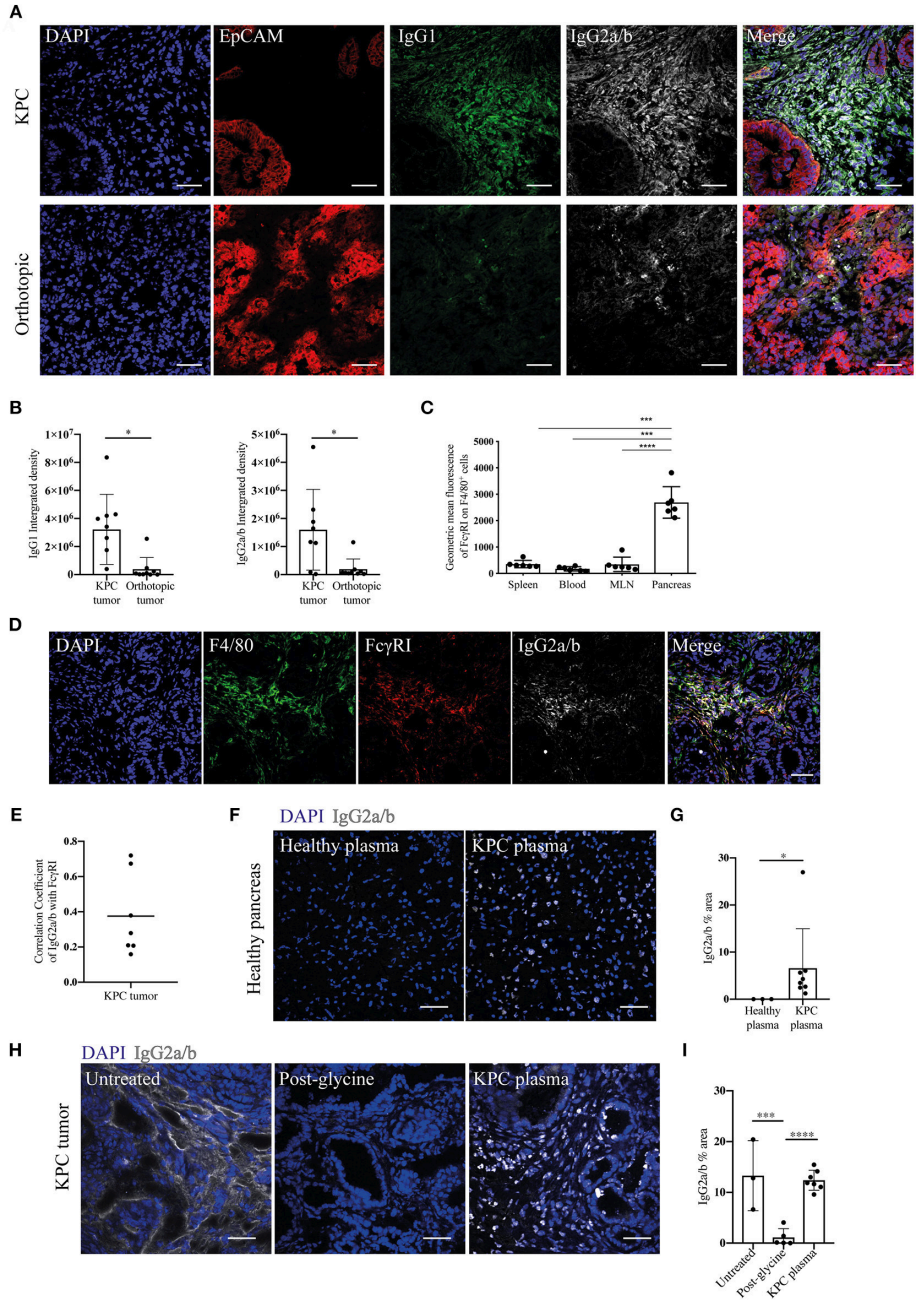
Evidence of immunoglobulin deposition in KPC tumors and IgG2a/b response against pancreatic antigens in KPC mice. **(A)** Representative immunofluorescence images of immunoglobulin deposition of IgG1 (green) and IgG2a/b (white) near EpCAM positive tumor cells (red) in KPC (*n* = 3) and orthotopic tumors (*n* = 4). **(B)** Quantification of the intensity (integrated density) of IgG1 and IgG2a/b (minus their respective isotype controls) in individual images of KPC and orthotopic tumors. **(C)** Flow cytometry analysis of geometric mean fluorescence of the marker FcɤRI on F4/80^+^ CD45^+^ cells in the spleen, blood, MLN and pancreas tumor of KPC mice. Each data point represents an individual mouse sample and error bars represent SD. Statistical significance was analyzed by unpaired *t*-test where ^*^p < 0.05, ^***^p < 0.001 and ^****^p < 0.0001. **(D)** Representative immunofluorescence images of colocalization of F4/80 (green), FcɤRI (red) and IgG2a/b (white) deposits, where DAPI (blue) was used as a nuclear marker in KPC tumors (*n* = 3). **(E)** Quantification of FcɤRI and IgG2a/b colocalization. The correlation coefficient between IgG2a/b and FcɤRI was calculated using ZEN software on individual images of KPC tumors, where a coefficient of 0 indicates no colocalization and 1 indicates complete colocalization. **(F)** Binding of plasma from healthy or KPC mice to sections of healthy pancreas, where IgG2a/b (white) was used to detect immunoglobulins and DAPI (blue) was used as a nuclear marker. **(G)** Quantification of the percentage area of IgG2a/b on individual images of pancreas sections incubated with either healthy or KPC plasma. **(H)** Immunofluorescence on KPC tumor sections stained for IgG2a/b (white): from left to right, native deposition in a KPC tumor, post-incubation with glycine to remove bound immunoglobulins, and post-incubation with glycine and then KPC plasma (*n* = 2). **(I)** Quantification of the percentage area of IgG2a/b on individual images of KPC tumor sections either untreated (showing native deposition in a KPC tumor), post-incubation with glycine to remove bound immunoglobulins and post-incubation with glycine and then KPC plasma. N refers to number of individual mouse tumors stained, mean and SD are also indicated. For all images scale bar is 50 μm.

Given the increase in IgGs in KPC pancreatic tumors, we wanted to both confirm this finding and compare this to orthotopic tumors. We found pronounced immunoglobulin deposition in KPC tumors (Figures 2A,B and Supplementary Figures S2A,B), in line with human data (27). In contrast, immunoglobulin deposition was markedly weaker to absent in orthotopic tumors (Figures 2A,B), suggesting a much-reduced B cell activation in this model that has not progressed to plasma cell differentiation. IgG2a is the only immunoglobulin subclass which can bind the FcɤRI as a monomer and not in an immune complex. Interestingly, we found macrophages upregulated FcɤRI at the tumor site (Figure 2C) and it colocalized with IgG2a/b deposition (Figures 2D,E), suggesting IgG2a binds as a monomer to FcɤRI on tumor-infiltrating macrophages in KPC tumors.

Previous analysis of PDAC patient serum revealed tumor-derived immunoglobulins recognize predominantly intracellular/nuclear antigens (32). In order to assess if this was also the case for KPC-derived Ig, we incubated sections of healthy pancreas with the plasma of either healthy or KPC mice. We found that only KPC-derived IgG2a/b bound strongly to nuclear components of healthy pancreatic cells (Figures 2F,G) and crucially not to cells in other organs (Supplementary Figure S2C). We repeated this assay on glycine-treated KPC tumors, in order to remove native immunoglobulins, and again found nuclear binding of KPC-derived IgG2a/b (Figures 2H,I). This suggested to us that a B cell response is mounted against pancreatic intracellular antigens during KPC PDAC, accurately recapitulating observations in human PDAC (32, 33).

Taken together, our data indicate B cells heavily infiltrate KPC tumors, become activated, enter the germinal center reaction and produce substantial immunoglobulin deposits at the tumor site. Crucially, germinal center B cells are observed mainly within or near the tumor in the draining lymph nodes but not in the spleen, suggesting a local B cell activation to tumor antigens. More importantly, the orthotopic model appear different, possibly because the faster tumor kinetics does not allow sufficient time to see a full B cell involvement in these tumors.

### Tumor-Infiltrating B Cells Acquire an Immunostimulatory Phenotype

The protumoral activity of B cells in other murine models has been partly attributed to their role as Bregs secreting cytokines IL-35 (46), IL-10 (47), and TGF-β (48), all described to induce immunosuppression. We found IL-10^+^ Bregs to represent only a small proportion of B cells infiltrating tumors (Figure 1H).

**Figure 3:**
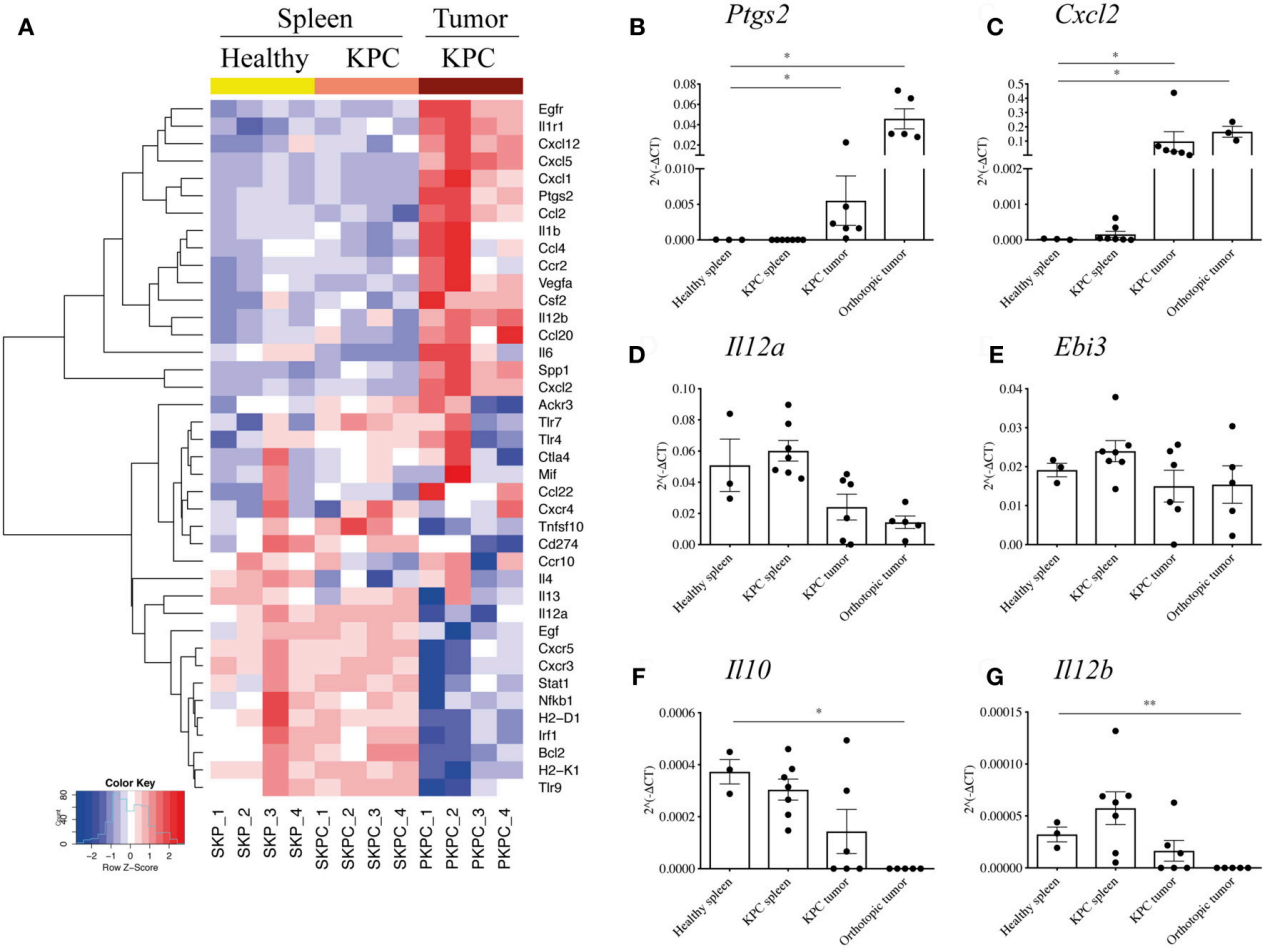
B cells acquire a proinflammatory phenotype within the pancreatic tumors. **(A)** Heat map constructed using row z-scores of log2-transformed gene expression values of significantly different genes in B cells analyzed with the RT^2^ Profiler™ PCR Array Mouse Cancer Inflammation & Immunity Crosstalk. B cells were isolated from healthy spleen – mice expressing only *Pdx-1-Cre* (yellow, SKP1-4), KPC spleens (salmon pink, SKPC1-4) and KPC tumors (brown, PKPC1-4). As the heat map shows, 4 samples per group were analyzed and normalized to *Gapdh*. **(B–G)** Validation of gene array and additional analysis by conventional qRT-PCR of 2^∧^(−ΔCt) values, normalized to housekeeping gene *Gapdh*, in B cells isolated from healthy spleen (*n* = 3), KPC spleen (*n* = 7), KPC tumor (*n* = 6), and orthotopic tumors (*n* = 3–5) in genes **(B)**, *Ptgs2*, **(C)** *Cxcl2*, **(D)** *Il12a*, **(E)** *Ebi3*, **(F)** *Il10*, and **(G)** *Il12b*. Each data point represents an individual sample, mean and SD are also indicated. Statistical significance was tested using Mann-Whitney test (non-parametric samples) and unpaired *t*-test (normally distributed samples) where ^*^p <0.05 and ^**^p < 0.01.

Therefore, to further investigate the phenotype of the large infiltration of B cells seen in KPC tumors and how they might influence the tumor microenvironment, we profiled the expression of 86 genes relating to cancer inflammation and immune cross-talk from tumor-infiltrating B cells, comparing them to splenic B cells both from KPC mice and healthy controls. Genes found to be significantly different are reported in the heatmap in Figure 3A.

Interestingly, splenic B cells showed a rather similar phenotype, whether derived from a KPC or healthy mouse, indicating the overall B cell population in the spleen largely retains its phenotype even in the presence of a pancreatic tumor, whereas B cells infiltrating the tumor develop a distinctly different profile. For the intra-tumoral B cells, there were numerous interesting features such as significantly upregulated proinflammatory cytokines (*Spp1, Il6, Csf2, Vegfa, Ccl4*), the inflammatory enzyme COX2 (*Ptgs2*), as well as chemokines involved in the recruitment of T cells in a germinal center (*Cxcl1, Cxcl2*, and *Cxcl5*) and macrophages/DCs (*Ccl2, Cxcl12, Ccl20*) (Figure 3A). On the other hand, genes associated with immunosuppression were downregulated, such as PDL1 (*Cd274*), as well as the IL-35 component *Il12a* (Figure 3A). The gene *Il10* was not upregulated in infiltrating B cells but rather expressed at low levels across all three groups (Supplementary Figure S3A).

Although the B cell infiltration is far weaker, we wanted to understand if orthotopic intratumoral B cells developed a different profile from KPC intratumoral B cells. Thus, a number of genes were selected for further qRT-PCR and compared against original and independent KPC samples with the addition of B cells from orthotopic tumors. Upregulation of *Ptgs2* and *Cxcl2* was confirmed in additional KPC tumor-derived B cells and was also observed in orthotopic B cells (Figures 3B,C). Although significantly downregulated in the array, we found a weak but not significant downregulation of *Il12a* in KPC and in orthotopic samples (Figure 3D). Additionally, *Ebi3*, which together with *Il12a* forms the immunosuppressive IL-35 (28), showed no upregulation in tumor B cells (Figure 3E). Taken together, these results indicate that KPC tumor B cells do not express IL-35. Furthermore, the immunosuppressive cytokine *Il10* was not upregulated in the array (Supplementary Figure S3A) or in additional KPC tumor B cells by qRT-PCR (Figure 3F), it was actually downregulated in orthotopic B cells (Figure 3F), further confirming the overall B-cell population within tumors holds a more proinflammatory and likely immunostimulatory phenotype. On the other hand, *Il12b* showed no difference in KPC samples analyzed by qRT-PCR and was weakly downregulated in orthotopic-derived B cells (Figure 3G), in contrast to the array which showed a certain level of upregulation (Figure 3A). It is not surprising that some results from an array are not subsequently validated (49, 50). This is why we further assessed important genes by qPCR and the results confirmed our initial findings.

Our data indicate that the PDAC tumor microenvironment uniquely shapes the phenotype of infiltrating B cells, demonstrated in KPC and similarly in orthotopic models. In both cases, infiltrating B cells possess a remarkably different phenotype to splenic B cells in tumor-bearing mice. This suggests a unique proinflammatory phenotype is acquired by B cells upon entry into the tumor microenvironment and the main difference between KPC and orthotopic tumors is likely to be the kinetics of this B-cell infiltration. Importantly, these results indicate that spleen-derived and tumor-derived B cells should not be used interchangeably in future studies.

### B Cell Depletion in Orthotopic PDAC Does Not Influence the Tumor-Immune Infiltrate

**Figure 4:**
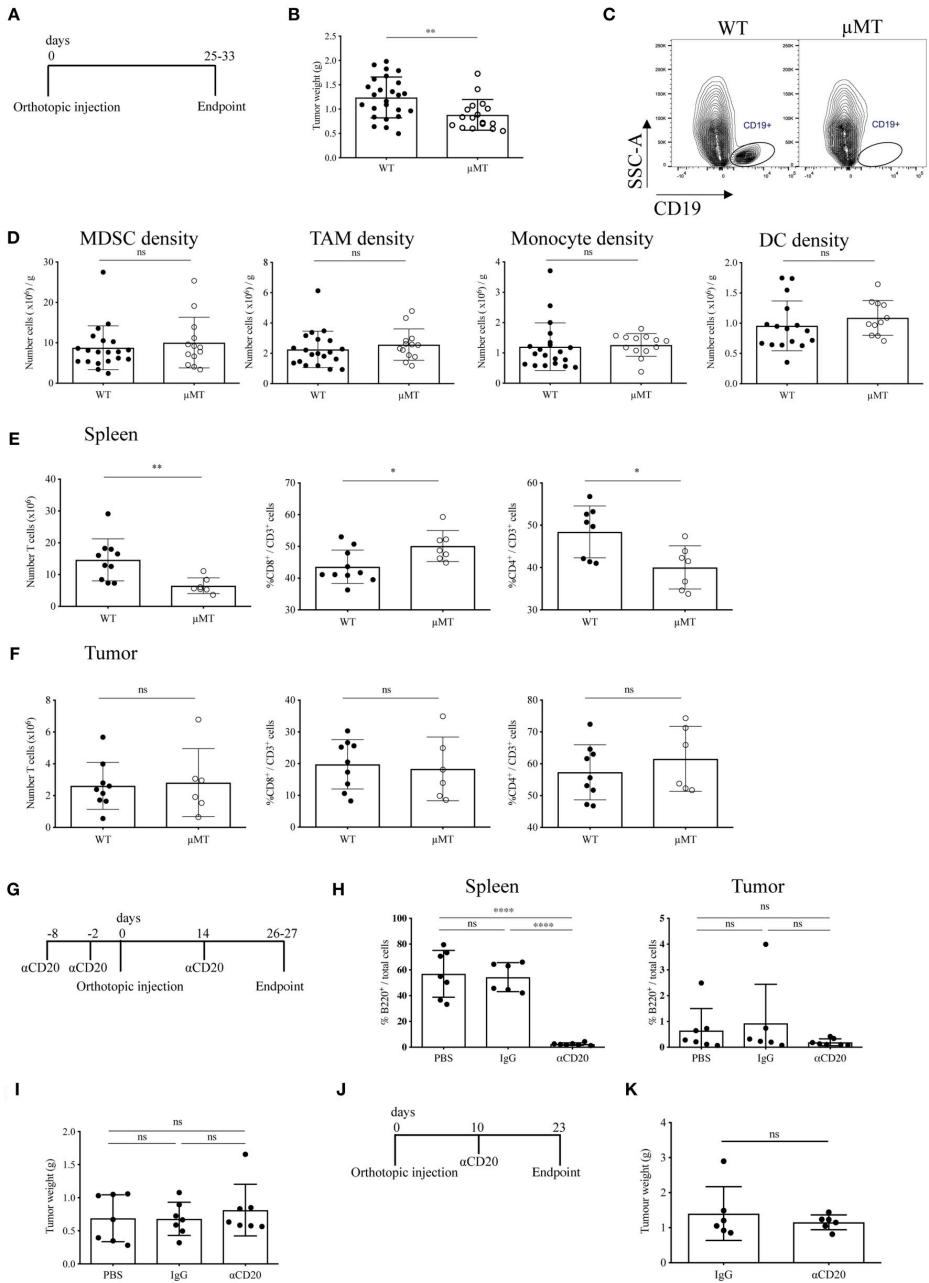
Two B-cell depletion strategies for orthotopic pancreatic tumors show different results. **(A)** Timeline of orthotopic tumor growth in WT and μMT^−/−^ mice. KPC-derived tumor cells were injected into the pancreas of WT and μMT^−/−^ littermates until tumors reached humane endpoint. **(B)** Gross orthotopic tumor weight at endpoint in WT (*n* = 25) and μMT^−/−^ (*n* = 17) littermates. **(C)** Flow cytometry of CD45^+^ CD19^+^ cells to confirm lack of B cells in μMT^−/−^ mice. **(D)** Density (number of cells per gram tissue) of myeloid cell populations (all gated on CD45^+^ CD11b^+^): myeloid-derived suppressor cells, MDSCs (Ly6G^+^ Ly6C^+^), tumor-associated macrophages, TAMs (Ly6G^−^ Ly6C^−^ F4/80^+^ MHC II^+^), monocytes (Ly6G^−^ Ly6C^hi^) and dendritic cells, DCs (Ly6G^−^ Ly6C^−^ F4/80^+^ MHC II^+^ CD11c^+^) analyzed by flow cytometry in WT and μMT^−/−^ tumors. **(E)** Number of T cells (CD45^+^ CD3^+^) (left) and proportion of CD8^+^ (middle) and CD4^+^ (right) in the spleen of orthotopic tumor-bearing WT and μMT^−/−^ mice. **(F)** Number of T cells (CD45^+^ CD3^+^) (left) and proportion of CD8^+^ (middle) and CD4^+^(right) in orthotopic tumors from WT and μMT^−/−^ mice. **(G)** Timeline of orthotopic tumor growth in B cell depleted mice. 250 μg of anti-CD20, IgG2a or PBS were injected i.v. at −8, −2 pre and 14 days post-orthotopic surgery. Mice were culled at endpoint at 26–27 days. *N* = 7 per group. **(H)** Percentage of B220^+^ cells out of total cells (as defined by all cells with a nucleus in the section) in sections of spleen (left) and tumor (right) in anti-CD20, IgG2a or PBS-treated mice (*n* = 7 per group). B220 was stained by IHC and cells analyzed using Definiens software. **(I)** Gross pancreatic tumor weight at endpoint in anti-CD20, IgG2a, or PBS-treated mice (*n* = 7 per group), where treatment was injected i.v. at −8, −2 pre and 14 days post-orthotopic surgery. **(J)** Timeline of orthotopic tumor growth in B cell depleted mice. 250 μg of anti-CD20 or IgG was injected i.v. at day 10 post-orthotopic surgery. Mice were culled at endpoint at day 23, with *n* = 6 per group. **(K)** Gross pancreatic tumor weight at endpoint in anti-CD20 or IgG2a-treated mice (*n* = 6 per group), where treatment was given at day 10 post-orthotopic surgery. Each data point represents an individual mouse, mean and SD are also indicated. Statistical significance was tested for **(B–F,K)** by unpaired *t*-test, for **(H)** (spleen) and **(I)** One-Way ANOVA and Bonferroni's post-test, for **(H)** (tumor) by Kruskal Wallis and Dunn's multiple comparison test, ^*^p < 0.05, ^**^p < 0.01, ^***^p < 0.0001.

Although in this study we have demonstrated a relative paucity of B cells in orthotopic tumors, it has been suggested that B cells may promote tumor growth in orthotopic models of PDAC (27, 28). To address this, we investigated the effect of B-cell loss in syngeneic orthotopic tumors through two methods; B-cell deficiency in μMT mice vs. transient B-cell depletion with anti-CD20 mAb. Injection of tumor cells in B-cell deficient, μMT mice (35) (Figure 4A) led to reduced tumor growth compared to WT littermates (Figure 4B), in agreement with others using similar PDAC cell lines and B-cell deficient models (27, 28). Owing to the loss of the B cell fraction (Figure 4C) and immunoglobulins (Supplementary Figure S4A), we then analyzed the tumor immune infiltrate and found comparable infiltration of MDSCs (CD45^+^, CD11b^+^, Ly6G^+^, Ly6C^+^), tumor associated macrophages (TAMs: CD45^+^, CD11b^+^, Ly6G^−^, Ly6C^−^, F4/80^+^, MHC II^+^), monocytes (CD45^+^, CD11b^+^, Ly6G^−^, Ly6C^hi^), and dendritic cell subsets (CD45^+^, CD11b^+^, Ly6G^−^, Ly6C^−^, F4/80^−^, MHC II^+^, CD11c^+^) within the tumor between μMT mice and WT littermates (Figure 4D). CD86 expression on TAMs and DCs, as well as TAM mannose receptor (MR) expression was also unchanged, indicating B-cell loss does not influence their polarization to a more immunomodulatory phenotype (Supplementary Figure S4B). Furthermore, the *ex vivo* cytokine production, degranulation activity and proportion of Tregs in the tumor-infiltrating T cell population was not altered (Supplementary Figure S4B). However, μMT mice have an altered immune system, with well-described skewing toward Th1 responses (51, 52). Indeed, in our experiments, we confirmed μMT mice with orthotopic tumors had a higher proportion of CD8^+^ to CD4^+^ T cells in the spleen (Figure 4E), whilst no change in T cell proportions in the tumor itself (Figure 4F).

Since our data indicated B cells to infiltrate orthotopic B cells poorly, we speculated that the reduced tumor growth observed in μMT mice was due to their intrinsically different immune system and especially their T cell phenotype. In order to confirm this finding, we used an alternative method to study B-cell loss in orthotopic tumor development. To this end, we depleted B cells in WT mice using an anti-CD20 antibody both before and during tumor progression (Figures 4G,J). We observed complete ablation of B cells at endpoint (Figure 4H and Supplementary Figure S4C). In contrast to our results obtained with μMT mice, B-cell depletion with anti-CD20 did not cause any reduction in tumor mass in either experimental setting (Figures 4I,K), further suggesting that intrinsic characteristics of B-cell deficient μMT mice may contribute to the effect on orthotopic tumor development, rather than the loss of B cells *per se*.

### B-Cell Depletion Does Not Slow Tumor Growth in KPC Mice

**Figure 5:**
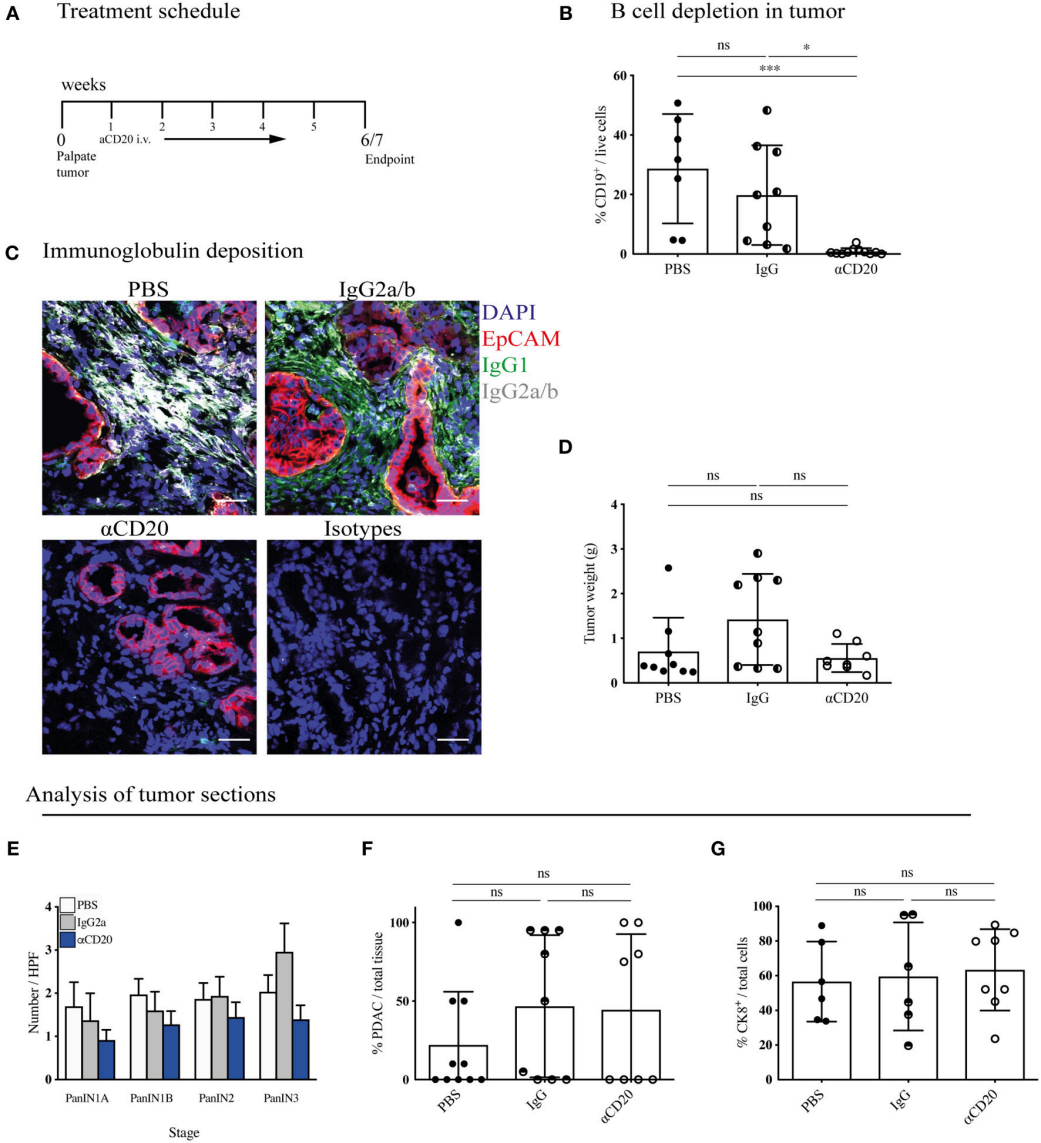
B-cell depletion does not slow tumor progression in KPC mice. **(A)** Treatment schedule of KPC mice with palpable tumors treated with either 200 μl PBS or 250 μg (resuspended in 200 μl PBS) of mouse IgG2a or mouse anti-CD20 i.v. once per week, for a total of 5 - 6 weeks. **(B)** Percentage of CD19^+^ cells out of total viable cells in KPC tumors analyzed by flow cytometry at the end of treatment course in anti-CD20 (*n* = 7), IgG2a (*n* = 9) or PBS-treated (*n* = 11) mice. **(C)** Representative immunofluorescence images of epithelial cell marker EpCAM (red), IgG1 (green), IgG2a/b (white), and nuclear marker DAPI (blue) in KPC tumors at the end of treatment course in anti-CD20 (*n* = 3), IgG2a (*n* = 2), or PBS-treated (*n* = 3) mice. **(D)** Gross pancreas weight at the end of treatment course in anti-CD20 (*n* = 11), IgG2a (*n* = 11) or PBS-treated (*n* = 8) mice. **(E)** Histological staging of PanINs where 10 high-power fields were analyzed per tumor section at 20X magnification for PBS- (*n* = 10), IgG- (*n* = 9), and anti-CD20-treated (*n* = 8) mice. **(F)** Histological analysis by a pathologist of percentage of PDAC out of total tissue in sections of KPC tumors for PBS- (*n* = 10), IgG- (*n* = 9), and anti-CD20-treated (*n* = 8) mice. **(G)** Quantification of percentage CK8-positive cells out of total cells (as defined by all cells with a nucleus in the section) in sections of KPC tumors for PBS- (*n* = 6), IgG2a- (*n* = 6) and anti-CD20-treated (*n* = 8) mice. Each data point represents an individual mouse, mean and SD are also indicated. **(B,E,G)** Significance was tested using One-Way ANOVA and Bonferroni's Multiple Comparison Test, and for **(D)**, **(F)** by Kruskal Wallis and Dunn's multiple comparison test, where ^*^p < 0.05 and ^***^p < 0.001.

Our data thus far indicated that, unlike in orthotopic PDAC, proinflammatory B cells heavily infiltrate KPC PDAC and these tumors display substantial deposition of immunoglobulins. If B cells exerted a protumor effect, then B cell deletion from KPC mice should result in reduced tumor growth. To this end, KPC mice with early stage, palpable PDAC tumors were treated for 5–6 weeks with B-cell depleting anti-CD20 antibody, in order to assess any potential therapeutic benefit (Figure 5A).

KPC tumors were then harvested and their immune infiltrate and tumor content analyzed. Treatment with anti-CD20 effectively depleted B cells in the pancreatic tumors (Figure 5B), spleen, MLN and blood (Supplementary Figure S5A). Plasma cells downregulate CD20 following differentiation, thus our treatment should not deplete pre-existing plasma cells. However, we found a significant decrease of plasma cells in the spleen (Supplementary Figure S5B), indicating that B cell depletion successfully blocked further plasma cell differentiation taking place during tumor progression. Accordingly, anti-CD20 treatment also strongly diminished immunoglobulin deposition within the tumor (Figure 5C and Supplementary Figures S5C,D). Nonetheless, tumor weight was not altered (Figure 5D). It is important to note that the KPC model is characterized by a highly heterogenous tumor growth, which varied independent of treatment. To overcome this heterogeneity, we randomly assigned mice to treatment arms and performed further histological analysis to determine if more subtle changes had occurred in response to B cell depletion. We assessed the prevalence of each PanIN subtype (Figure 5E) or percentage of PDAC content (Figure 5F and Supplementary Figure S5E), which also revealed no difference between treatment groups. Further staining for the epithelial marker cytokeratin 8 (CK8) confirmed this result (Figure 5G and Supplementary Figure S5F). We also analyzed the tumor microenvironment and found that the infiltration of T cells and myeloid subsets into the tumor was unchanged by B-cell depletion, as measured by flow cytometry (Supplementary Figure S5G). Taken together, these data indicate that B-cell depletion does not lead to reduced tumor growth in the more relevant spontaneous KPC model, demonstrating B cells do not drive PDAC progression.

## Discussion

In the present study, we sought to clarify the relevance of two widely used models in PDAC research: the KPC and the orthotopic to dissect the role of B cells in the tumor microenvironment. It is well-described that both the kinetics and stromal content of the two models differ significantly. A longer disease progression (23) and pronounced stromal reaction is found in KPC, as in human PDAC tumors, whereas accelerated progression (25) and dominant malignant cell component is found in orthotopic tumors (26). This is important, as the stroma is a salient feature of PDAC (53) and crucially the main site for B cell localization (54). In line with this, we show B cells are major components of KPC tumors compared to their relative paucity in orthotopic tumors, suggesting that orthotopic tumors are not suitable to study the role played by these immune cells in pancreatic cancer. In a previous publication, B cell depletion was assessed in the KC model but as anti-CD20 was administered from 2 to 10 weeks of age (29), this was likely preceding disease initiation, since KC mice do not develop PanINs until after 8 weeks and invasive disease after 5 months (24). Tumor latency may be an important factor in B cell recruitment, it is unlikely that in early stage KC mice or in orthotopic models, B cells would have had sufficient time to infiltrate, experience antigens and be activated by the tumor microenvironment. In this regard, models where B cells do not form a substantial component of the tumor may instead only reflect the contribution of B cells within the secondary lymphoid organs on tumor initiation or progression.

In other cancers, the contribution of B cells to a favorable prognosis has been attributed to their production of immunoglobulins in response to antigen, which aid the antitumor response (13, 17, 18), as well as their ability to stimulate a T cell response through antigen presentation (55, 56). We also found clear evidence of B cell activation, germinal center entry, plasma cell expansion and immunoglobulin production in KPC mice, yet conversely found minimal signs of this in orthotopic mice. The presence of B cells within TLS confers improved survival in PDAC, and TLS have been shown to be site of germinal center activation, together, these results suggest a positive role for B cell activation in the antitumoral response (20). Also B cell infiltrates observed by us in KPC tumors appeared in clusters in the stroma. It is known that immunoglobulins produced during human PDAC predominantly recognize intracellular or intranuclear antigens (32). Mirroring human disease, we confirm that this also occurs in KPC mice, with a strong IgG2a/b response against intranuclear pancreatic antigens. The exact antigens recognized by the immunoglobulins were not assessed in this study. Normally, B cells are tolerized against extracellular self-antigens and thus would not commonly react against such markers, or at least not in large numbers. However, the large release of intracellular content during cell death that occurs in tumors allows B cells to circumvent this tolerization and react against these newly exposed antigens. Indeed, this takes place in other cancers, where B cells mount immunoglobulin responses against proteins such as p53 (31) and β-actin (57). Similarly, the production and deposition of autoantibodies recognizing intracellular targets has been shown in ovarian cancer patients (18). It is possible to hypothesize that nuclear antigens become available as tumor cells die. While Igs directed against such antigens may not target living tumor cells for degradation, they can nonetheless aid the activation of other immune cells such as dendritic cells (18). It is unclear if this antigen recognition shapes the anti-PDAC immune response in our setting and further studies are required to clarify this point. Nonetheless, our results show that deposited immunoglobulins are not present in immune complexes and therefore are not likely to influence myeloid cells via this route in pancreatic cancer.

Regarding the phenotype of tumor-infiltrating B cells, there is little description in the literature, except for the production of immunosuppressive cytokines by small B cell subsets, in which splenic B cells are often utilized as representative of tumor-infiltrating B cells (3, 28, 58, 59). We are the first to isolate tumor-infiltrating B cells as a whole population in order to assess their overall phenotype. Interestingly, we found intratumoral B cells upregulate the chemokines *Cxcl1, Cxcl2* and *Cxcl5*, which together are the murine equivalent of CXCL8/IL-8. A previous study has shown that B cells are the primary source of CXCL8 when present within a GC (60). T cells within the GC upregulate CXCR1, the receptor for CXCL8, thus suggesting B cells use the chemokine to recruit T cells to support the GC reaction. Previously, B-cell rich TLS within PDAC have also demonstrated high expression of CXCL8, indicating it might be important for the generation of TLS within tumors (20). Furthermore, CXCL8 is also upregulated in tumor-derived B cells isolated from ovarian cancer patients (18), in which B cells are present in TLS and also associate with improved prognosis.

Notably, we document the upregulation of many proinflammatory genes by B cells from within the KPC tumor compared to splenic-derived B cells. This is the first evidence that points to a role other than an immunosuppressive one by tumor-associated B cells. Correlative studies from human patient samples would suggest the inflammatory phenotype acquired by B cells within tumors might be helping the anti-tumor response (20, 30). We hypothesize that B cells produce the mediators not in the vicinity of tumor cells but rather within the tumor stroma, as our B cell staining indicates. Within the stroma, B cells are more likely to affect the phenotype of adjacent immune cells, rather than tumor cells themselves. It is known that some of the mediators expressed by B cells can have an immunostimulatory role, for example, Ccl20 is important in DC recruitment and CD8^+^ T cell cross-priming (61), whereas PGE2 (downstream product of COX2) (62) and Osteopontin (*Spp1*) (63) can both stimulate DC maturation and aid a Th1 response.

Interestingly, we observed a similar phenotype in orthotopic-derived B cells, demonstrating that B cells may be activated similarly when entering the PDAC-microenvironment, independent of model. However, it is likely that the timeframe of the orthotopic model does not allow the recruitment and differentiation to occur to the same extent as in KPC, therefore the effect that B cells exert on tumor progression in orthotopic tumors is probably rather marginal. Whereas others reported the production of immunosuppressive cytokines in different conditions and B cell subpopulations (3, 28, 58, 64), we did not detect an upregulation of genes associated with immunosuppression in tumor-derived B cells, namely *Il-12a, Ebi3* (which together form IL-35) and *Il-10*. We found a small percentage of IL-10^+^ Bregs within the tumor, however, they seemed to be in the minority, when considering the whole B cell population infiltrating PDAC tumors. We hypothesize that Bregs subpopulations may play a more significant role in secondary lymphoid organs such as the spleen and their removal might slow tumor initiation. However, once a tumor is established and B cells begin infiltrating, their phenotype is shaped by the tumor environment in a direction that supports T and myeloid cell recruitment, antigen presentation and T cell priming. Therefore, their removal at this stage would not be beneficial. This is corroborated by our finding that B cell depletion in KPC mice carrying palpable tumors does not alter tumor growth. If B cells played a protumorigenic role once tumors were established, their depletion should cause a decrease in tumor growth. However, we assessed tumor progression in multiple ways but none of the methods used showed a difference after B cell loss. On the other hand, within the same time frame, IgG treatment showed a trend for increased tumor growth, as previously reported (29). If B cells were helping the anti-tumor immune response, their removal might actually be detrimental and increase tumor growth in the long term. This remains to be established, for example with a longer treatment with anti-CD20 in KPC mice. In our experimental setting, we were not able to extend the number of anti-CD20 injections for a longer time frame and on a larger cohort of mice but these experiments would be important to fully understand the effects exerted by B cells in human PDAC.

## Concluding Remarks

We highlight in this study the critical importance of using relevant murine models in assessing the tumor immune infiltrate of pancreatic cancer. In particular B cells, which depend on stroma in order to infiltrate PDAC tumors, demonstrate vastly different recruitment and activation between the KPC and orthotopic model of PDAC. We additionally show that, unlike in the spleen, B cells infiltrating the tumor acquire a distinctly proinflammatory phenotype and their depletion in KPC with small but detectable tumors has no effect of tumor progression. Our data and that collected by others suggest a multifaceted role for B cells in tumor establishment and progression, with a more immunosuppressive role ascribed to B cells in secondary lymphoid organs, likely important at tumor inception, and a more immunostimulatory one once B cells start to be recruited in the tumor environment. This suggests B cells might be important components of the tumor microenvironment in instigating an antitumoral response and this should be considered when designing new immunotherapies for pancreatic cancer.

## Data Availability

The datasets generated for this study are available on request to the corresponding author.

## Author Contributions

SS and MC: conception, design, and methodology; SS, JC, JM, CG, EM, SB, FB, HK, and MC: acquisition of data (provided animals, acquired and managed patients, and provided facilities); SS, CG, EM, and MC: analysis and interpretation of the data (e.g., statistical analysis, biostatistics, and computational analysis); SS, JC, JM, CG, EM, SB, FB, HK, and MC: writing and reviewing the manuscript; MC: study supervision.

### Conflict of Interest Statement

The authors declare that the research was conducted in the absence of any commercial or financial relationships that could be construed as a potential conflict of interest.

## Supplementary Material

Supplementary Figure S1 Analysis of B cells in murine PDAC. **(A)** Representative flow cytometry gating strategy to exclude debris, doublets, and non-viable cells (FVD, fixable viability dye). B cells were gated as CD45^+^ CD19^+^. **(B)** KPC tumor sections were stained with B220 (red) as a marker of B cells and E-Cadherin (white) as an epithelial and tumor cell marker. DAPI was used as a nuclear marker (blue). *N* = 4. Images were taken at 40X and scale bar represents 50 μm. **(C)** Immunohistochemistry images of B cells using B220 (brown) in orthotopic tumors (*n* = 5), where a section of spleen was used as a positive control (*n* = 1). Images shown are 2X and scale bar is 1,000 μm. **(D)** Proportion of B cells (CD19^+^) and T cells (CD3^+^) out of CD45^+^ cells in orthotopic tumors (*n* = 9). The association was assessed using a Pearson correlation. **(E)** Gating strategy for CD95^+^ on GL7^hi^ CD19^+^ B cells, where an FMO was used as a gating control. **(F)** Gating strategy to confirm presence of germinal center B cells as CD45^+^ CD19^+^ CD38^−^ CD95^+^ cells. **(G)** Gating strategy to define CD11b^+^ B220^+^ B1 B cells. **(H)** Gating strategy for IgG1 on CD19^+^ B cells, where an FMO was used as a gating control. **(I)** Gating strategy for Ig2a/b on CD19^+^ B cells, where an FMO was used as a gating control. **(J)** Gating strategy for IgG3 on CD19^+^ B cells, where an FMO was used as a gating control. **(K)** Gating strategy for IgA on CD19^+^ B cells, where an FMO was used as a gating control. **(L)** Gating strategy for IgE on CD19^+^ B cells, where an FMO was used as a gating control. **(M)** Gating strategy for intracellular IL-10 on B220^+^/CD19^+^ B cells, which had been stimulated *ex vivo* with LPS, PMA and Brefeldin. **(N)** Flow cytometry quantification of proportion of CD138^hi^ plasma cells out of total CD45^+^ immune cells in the pancreas of healthy (*n* = 6) and tumor of KPC (*n* = 7) mice. **(O)** Flow cytometry quantification of proportion of CD138^lo^ plasmablasts out of total CD45^+^ immune cells in the spleen of healthy (*n* = 9) and KPC (*n* = 12) mice. **(P)** Flow cytometry quantification of proportion of CD138^hi^ plasma cells out of total CD45^+^ immune cells in the spleen of aged-matched mice, either injected orthotopically with tumor cells (black), Matrigel only (red), or healthy controls (blue). Mice were culled at 7, 14, and 21 days post-injection. **(Q)** Relative concentration of C1q-Ig immune complexes as measured by ELISA in healthy control serum (*n* = 3) and KPC serum (*n* = 5). Negative and positive controls were provided by the ELISA kit manufacturer, dashed line represents threshold for positivity. A confirmation test that disrupts ICs was performed for each sample, as recommended by the manufacturer, however, results show no difference since no ICs were detected. Each data point represents an individual mouse, mean and SD are also indicated. Statistical significance was tested for by unpaired *t*-test.

Supplementary Figure S2 Immunoglobulin response in KPC mice. **(A)** Representative immunofluorescence images of immunoglobulin deposition of IgG1 (green) and IgG2a/b (white) near EpCAM positive pancreatic cells (red) where DAPI (blue) was used as a nuclear marker in healthy pancreas and isotype controls. The same settings were used for acquiring images from healthy and tumor tissues. **(B)** Representative immunofluorescence images of immunoglobulin deposition in KPC tumors (*n* = 2) of IgG3 (green) and IgM (white) where EpCAM (red) was used to stain tumor/epithelial cells and DAPI (blue) was used as a nuclear marker. **(C)** Sections of healthy kidney, liver, lung and muscle were incubated with control (healthy) and KPC plasma to determine binding of immunoglobulin to non-pancreas cells. Slides were then stained for IgG2a/b (white) and DAPI was used as a nuclear marker (blue). All images were taken with a 40X objective and the scale bar represents 50 μm.

Supplementary Figure S3 No upregulation of immunosuppressive cytokine Il10 in KPC tumor-derived B cells. **(A)** Gene expression of *Il10* in B cells isolated from healthy spleen (*Pdx-1Cre*), and the spleen and tumor of KPC mice. Gene expression was measured on the RT^2^ Profiler™ PCR Array Mouse Cancer Inflammation & Immunity Crosstalk. The expression is normalized to the house keeping gene *Gapdh* and expressed as 2^∧^(−ΔCt) values. Each data point represents an individual mouse (*n* = 4) and statistical significance was tested using Mann-Whitney test.

Supplementary Figure S4 The effect of B cell depletion in orthotopic PDAC. **(A)** Representative immunofluorescence images of absent immunoglobulin deposition of IgG1 (green channel) and IgG2a/b (white channel) near EpCAM positive tumor cells (red) where DAPI (blue) was used as a nuclear marker in μMT^−/−^ tumors (*n* = 6). **(B)** Flow cytometry analysis of tumors of WT and μMT^−/−^ mice. Upper panel from left to right, percentage of CD86^+^ TAMs (CD45^+^ CD11b^+^ Ly6G^−^ Ly6C^−^ F4/80^+^ MHC II^+^), CD86^+^ DCs (CD45^+^ CD11b^+^ Ly6G^−^ Ly6C^−^ F4/80^−^ MHC II^+^ CD11c^+^), and CD206/Mannose receptor (MR)^+^ TAMs. Middle panel: *ex vivo* characterization of T cells from tumors of WT and μMT^−/−^ mice following stimulation: from left to right, Ki67^+^ proliferation, IFNɤ^+^ and TNFa^+^ in CD8^+^ T cells (upper panels) and CD4^+^ T cells (lower panels), with the additional analysis of FOXP3^+^ Tregs. Each data point represents an individual mouse, mean and SD are also indicated. Statistical significance was tested using an unpaired *t*-test. **(C)** Representative IHC images of B220^+^ B cells (brown) on spleen and tumor sections from mice harboring orthotopic tumors treated with PBS (*n* = 7), IgG (*n* = 6), or anti-CD20 (*n* = 7), injected i.v. at −8, −2 pre and 14 days post-orthotopic surgery. Mice were culled at endpoint at 26–27 days. The scale bar represents 200 μm.

Supplementary Figure S5 Characterization of the effects of anti-CD20 B-cell depletion in KPC mice. **(A)** Flow cytometry analysis of the percentage of CD19^+^ cells out of CD45^+^ cells in the spleen, mesenteric lymph node and blood of KPC mice treated with PBS (*n* = 5), IgG2a (*n* = 5) or anti-CD20 (*n* = 5–7). **(B)** Flow cytometry of CD138^hi^ plasma cells out of total live cells in the spleen of KPC mice treated with anti-CD20 (*n* = 4) compared to IgG2a-treated controls (*n* = 3). **(C)** Representative immunofluorescence images of immunoglobulin deposition of IgG1 (green) and IgG2a/b (white) near EpCAM positive epithelial/ tumor cells (red) where DAPI (blue) was used as a nuclear marker in KPC tumors following treatment with PBS, IgG2a of anti-CD20. Scale bar represents 50 μm. **(D)** Quantification of the percentage area of IgG1 and IgG2a/b (or respective isotype controls) on individual images of KPC tumor sections from mice that had been treated with either PBS, IgG2a or anti-CD20. Significance was tested using a One-Way ANOVA and *post-hoc* Tukey's multiple comparison test. **(E)** Representative hematoxylin stained sections of KPC tumors following treatment with PBS, IgG2a and anti-CD20, which were used to stage PanINs and percentage of PDAC per section. Scale bar is 1,000 μm. **(F)** Representative image of CK8 epithelial staining of KPC tumors from PBS- (*n* = 6), IgG- (*n* = 6), and anti-CD20-treated (*n* = 8) mice compared to isotype control. Scale bar is 200 μm. **(G)** Flow-cytometry analysis of immune cells isolated from tumors of PBS- (*n* = 6–8), IgG- (*n* = 4–9) and anti-CD20-treated (*n* = 4–11) mice. Graphs on upper panels from left to right show absolute number of CD3^+^ T cells, percentage of CD4^+^ cells out of CD3^+^ cells, percentage of CD8^+^ cells out of CD3^+^ cells, percentage of FOXP3^+^ (Tregs) out of CD3^+^CD4^+^. Graphs on lower panel from left to right show absolute number of CD11b^+^ (myeloid) cells, percentage of Ly6G^+^Ly6C^+^ (MDSCs) cells out of CD11b^+^ cells, percentage of Ly6G^−^Ly6C^+^ cells (monocytes) out of CD11b^+^ cells, percentage of F4/80^+^MHC II^+^ cells (TAMs) out of CD11b^+^ cells. Each data point represents an individual mouse, mean ± SD are also indicated. Statistical significance was tested using a One-Way ANOVA and Bonferroni's post-test, where ^*^*p* < 0.05 and ^****^*p* < 0.0001.

Supplementary Table S1 Antibodies used for flow cytometry.

Supplementary Table S2 Antibodies used in immunofluorescence and immunohistochemistry.

Supplementary Table S3 Isotype antibody controls used in immunofluorescence and immunohistochemistry.

Supplementary Table S4 Secondary antibodies used in immunohistochemistry.
