# Supplementary material for: Discrepancies in the Tumor Microenvironment of Spontaneous and Orthotopic Murine Models of Pancreatic Cancer Uncover a New Immunostimulatory Phenotype for B Cells
Source: Front Immunol. 2019 Mar 27;10:542. doi: 10.3389/fimmu.2019.00542 (PMC6445859; doi:10.3389/fimmu.2019.00542)

Supplementary Figure 1 A-D

A

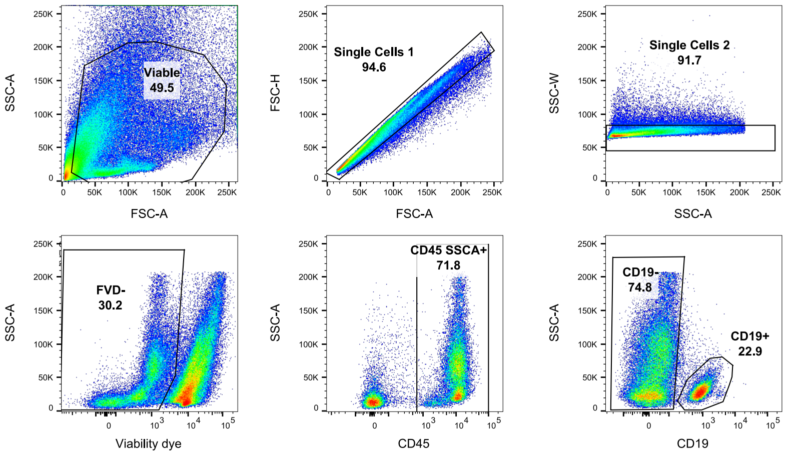

B

KPC tumor E-Cadherin low area

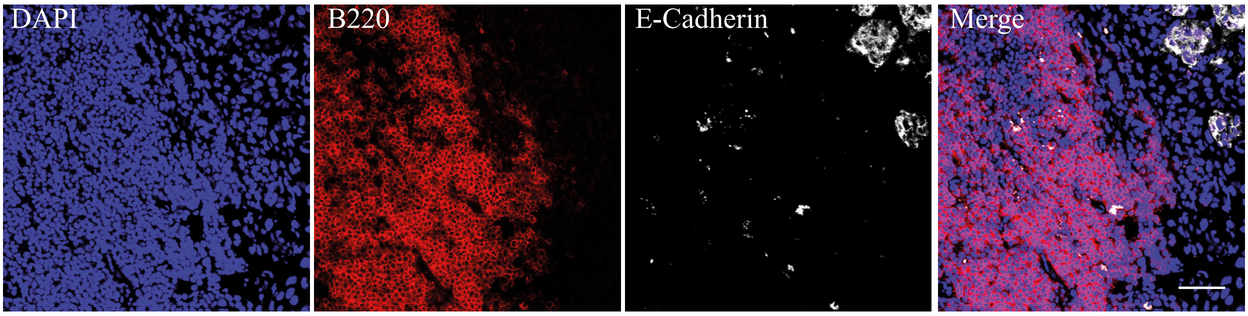

KPC tumor E-Cadherin rich area

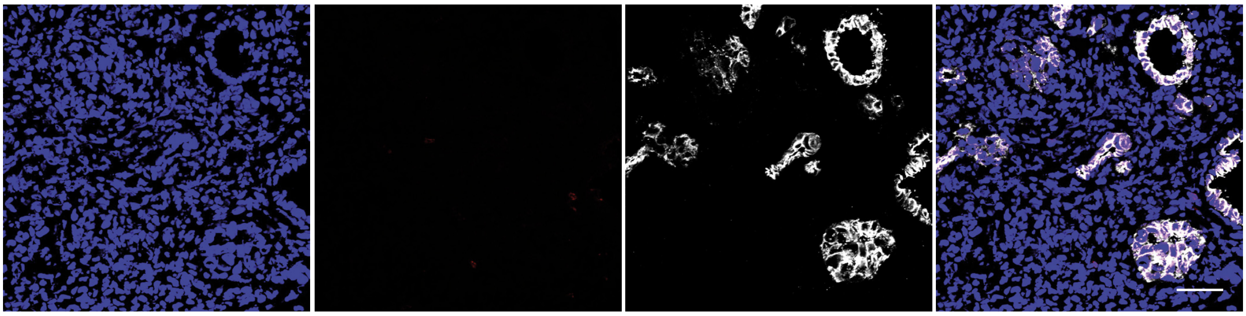

C

B220 in orthotopic tumor

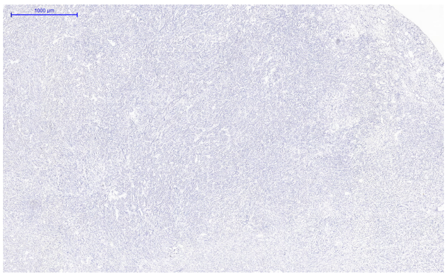

Positive control: B220 in spleen

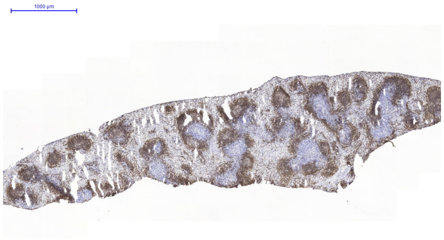

D

Orthotopic tumors

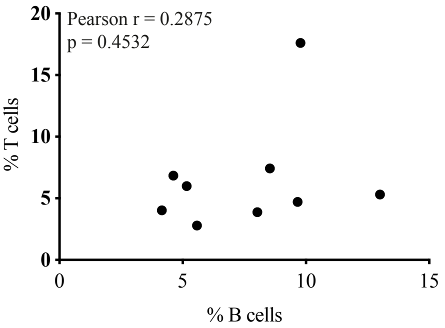

Supplementary Figure 1 E-Q

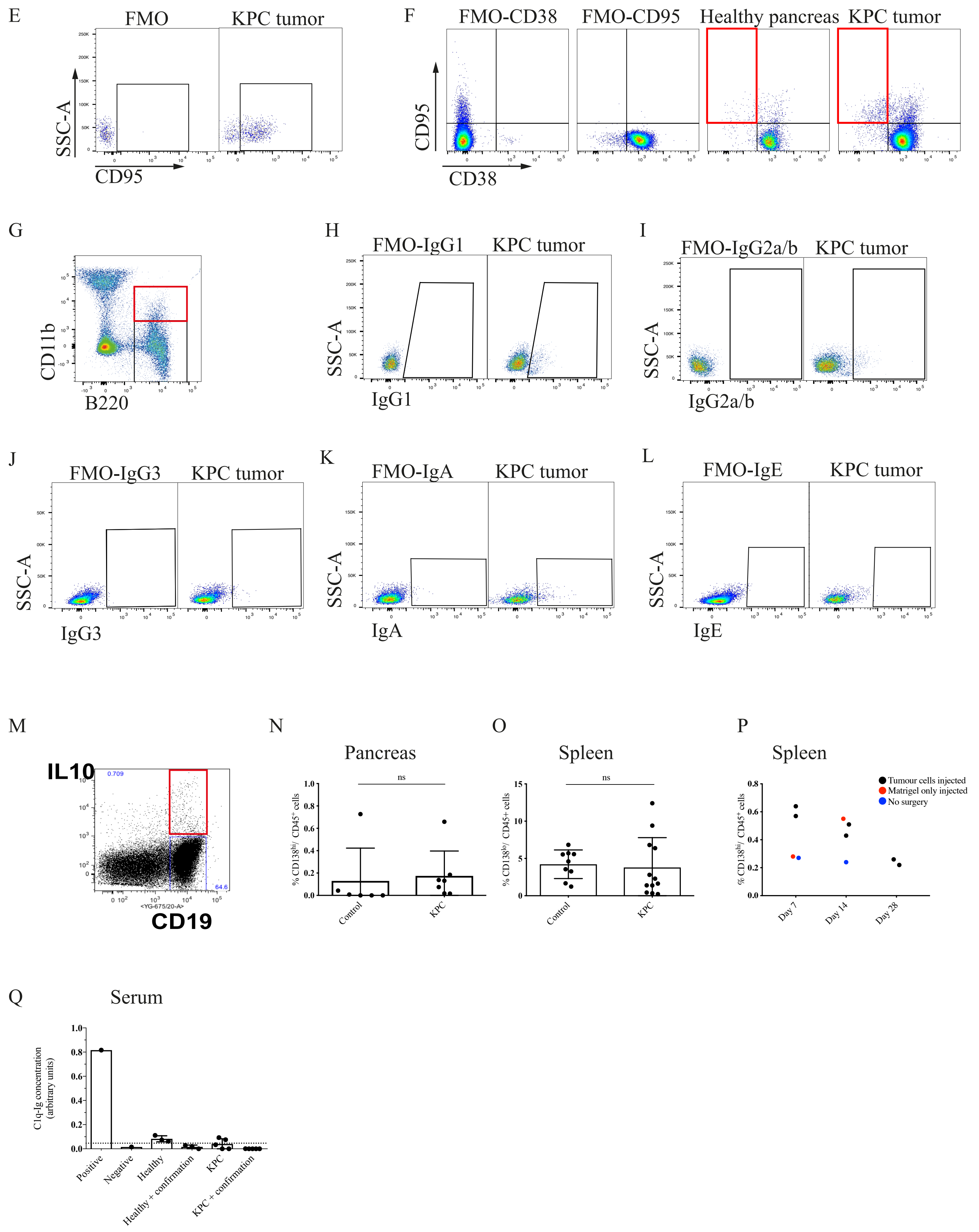

Supplement: Supplementary Figure S1 — Analysis of B cells in murine PDAC. (A) Representative flow cytometry gating strategy to exclude debris, doublets, and non-viable cells (FVD, fixable viability dye). B cells were gated as CD45+ CD19+. (B) KPC tumor sections were stained with B220 (red) as a marker of B cells and E-Cadherin (white) as an epithelial and tumor cell marker. DAPI was used as a nuclear marker (blue). N = 4. Images were taken at 40X and scale bar represents 50 μm. (C) Immunohistochemistry images of B cells using B220 (brown) in orthotopic tumors (n = 5), where a section of spleen was used as a positive control (n = 1). Images shown are 2X and scale bar is 1,000 μm. (D) Proportion of B cells (CD19+) and T cells (CD3+) out of CD45+ cells in orthotopic tumors (n = 9). The association was assessed using a Pearson correlation. (E) Gating strategy for CD95+ on GL7hi CD19+ B cells, where an FMO was used as a gating control. (F) Gating strategy to confirm presence of germinal center B cells as CD45+ CD19+ CD38− CD95+ cells. (G) Gating strategy to define CD11b+ B220+ B1 B cells. (H) Gating strategy for IgG1 on CD19+ B cells, where an FMO was used as a gating control. (I) Gating strategy for Ig2a/b on CD19+ B cells, where an FMO was used as a gating control. (J) Gating strategy for IgG3 on CD19+ B cells, where an FMO was used as a gating control. (K) Gating strategy for IgA on CD19+ B cells, where an FMO was used as a gating control. (L) Gating strategy for IgE on CD19+ B cells, where an FMO was used as a gating control. (M) Gating strategy for intracellular IL-10 on B220+/CD19+ B cells, which had been stimulated ex vivo with LPS, PMA and Brefeldin. (N) Flow cytometry quantification of proportion of CD138hi plasma cells out of total CD45+ immune cells in the pancreas of healthy (n = 6) and tumor of KPC (n = 7) mice. (O) Flow cytometry quantification of proportion of CD138lo plasmablasts out of total CD45+ immune cells in the spleen of healthy (n = 9) and KPC (n = 12) mice. (P) Flow cytometry quantif [file Image_1.pdf]
