# Supplementary figures and images for: Discrepancies in the Tumor Microenvironment of Spontaneous and Orthotopic Murine Models of Pancreatic Cancer Uncover a New Immunostimulatory Phenotype for B Cells
Source: Front Immunol. 2019 Mar 27;10:542. doi: 10.3389/fimmu.2019.00542 (PMC6445859; doi:10.3389/fimmu.2019.00542)

Supplementary Figure 2

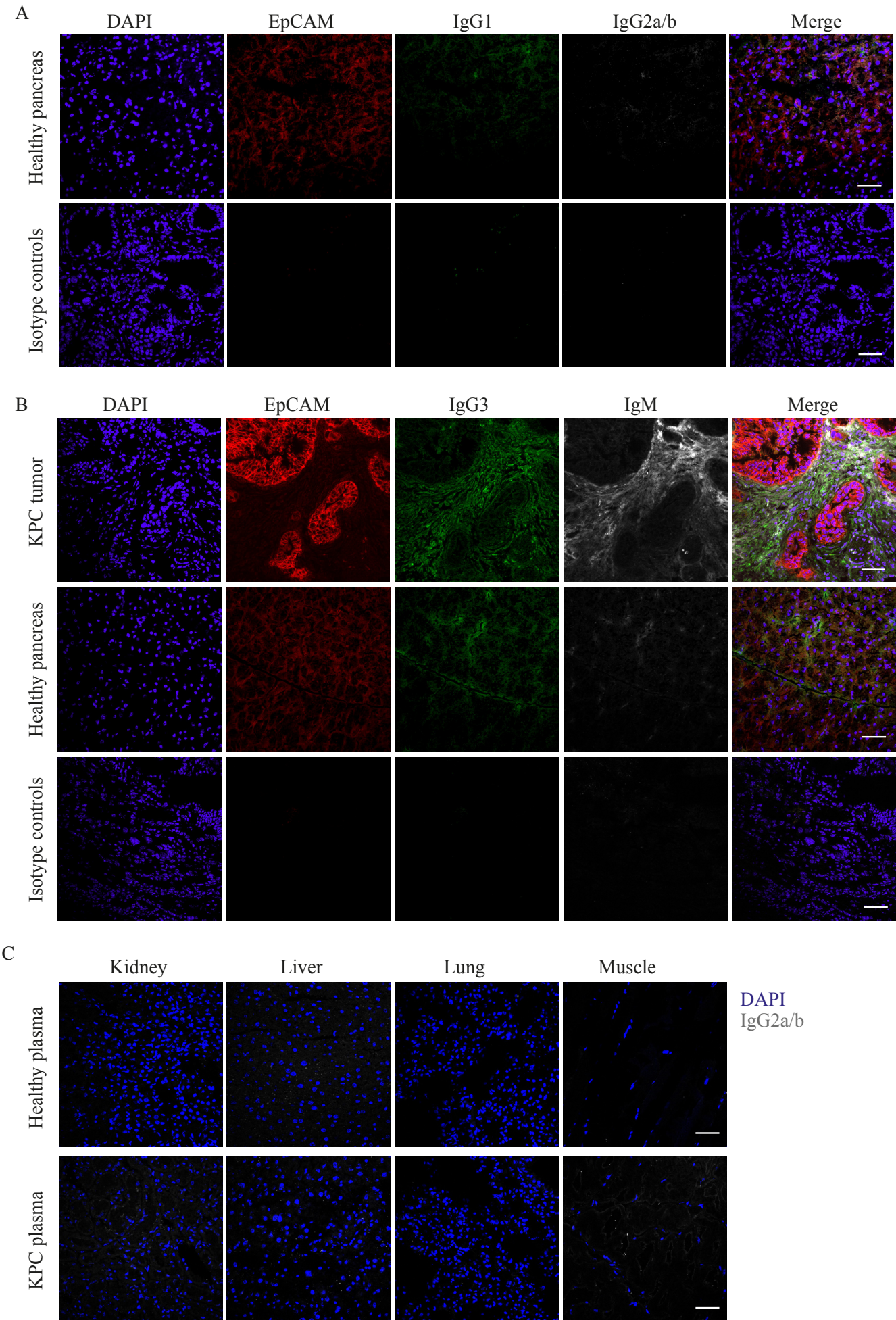

Supplement: Supplementary Figure S2 — Immunoglobulin response in KPC mice. (A) Representative immunofluorescence images of immunoglobulin deposition of IgG1 (green) and IgG2a/b (white) near EpCAM positive pancreatic cells (red) where DAPI (blue) was used as a nuclear marker in healthy pancreas and isotype controls. The same settings were used for acquiring images from healthy and tumor tissues. (B) Representative immunofluorescence images of immunoglobulin deposition in KPC tumors (n = 2) of IgG3 (green) and IgM (white) where EpCAM (red) was used to stain tumor/epithelial cells and DAPI (blue) was used as a nuclear marker. (C) Sections of healthy kidney, liver, lung and muscle were incubated with control (healthy) and KPC plasma to determine binding of immunoglobulin to non-pancreas cells. Slides were then stained for IgG2a/b (white) and DAPI was used as a nuclear marker (blue). All images were taken with a 40X objective and the scale bar represents 50 μm. [file Image_2.pdf]

Supplementary Figure 3

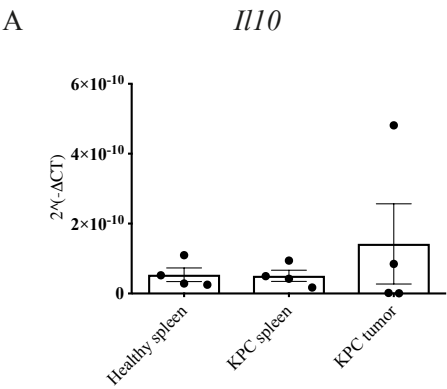

Supplement: Supplementary Figure S3 — No upregulation of immunosuppressive cytokine Il10 in KPC tumor-derived B cells. (A) Gene expression of Il10 in B cells isolated from healthy spleen (Pdx-1Cre), and the spleen and tumor of KPC mice. Gene expression was measured on the RT2 Profiler™ PCR Array Mouse Cancer Inflammation & Immunity Crosstalk. The expression is normalized to the house keeping gene Gapdh and expressed as 2∧(−ΔCt) values. Each data point represents an individual mouse (n = 4) and statistical significance was tested using Mann-Whitney test. [file Image_3.pdf]

Supplementary Figure 4

A

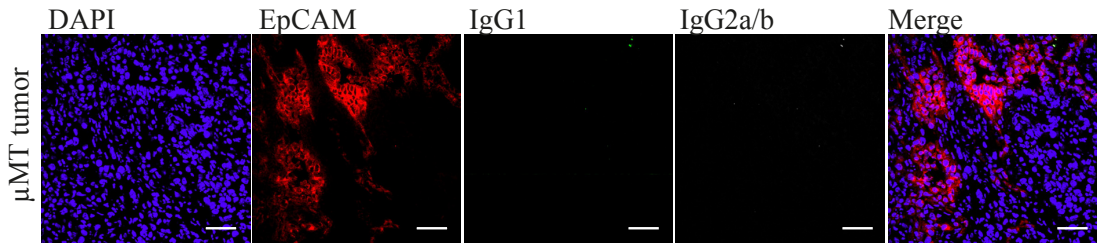

B

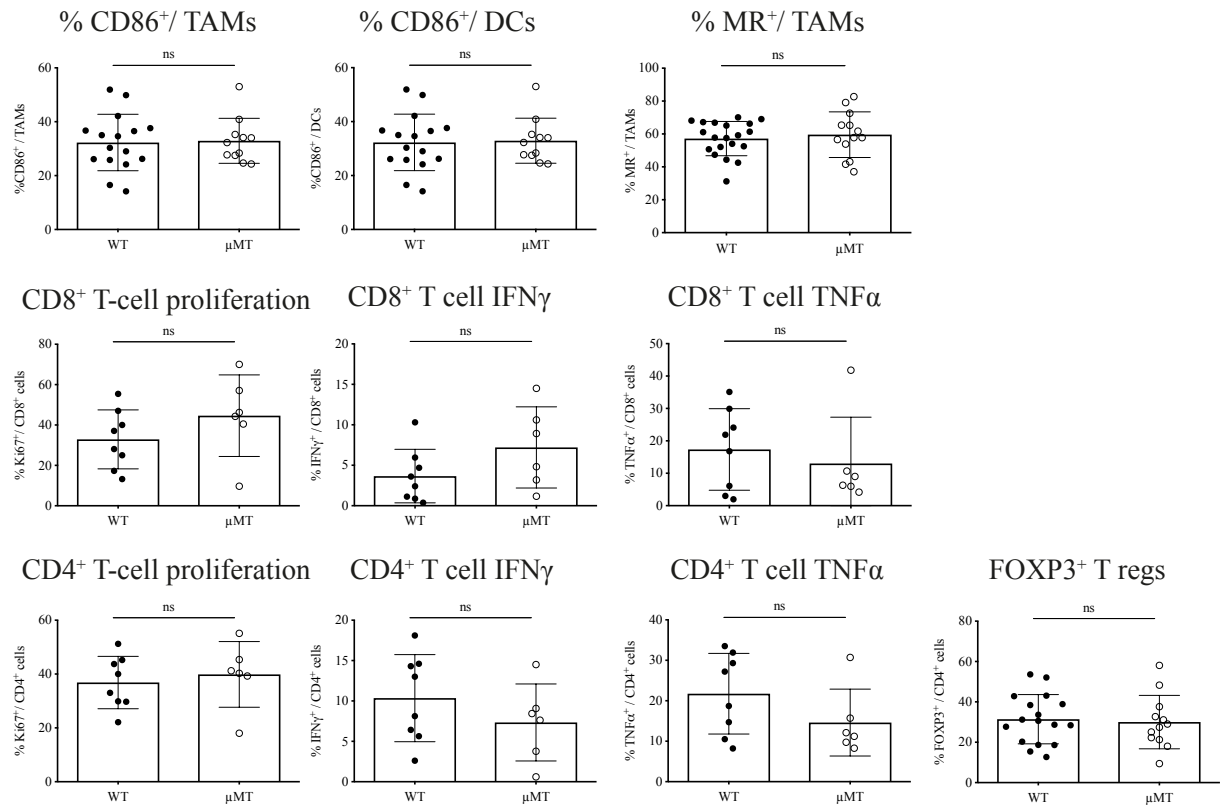

C B220 staining (brown) in anti-CD20 treated orthotopic tumors

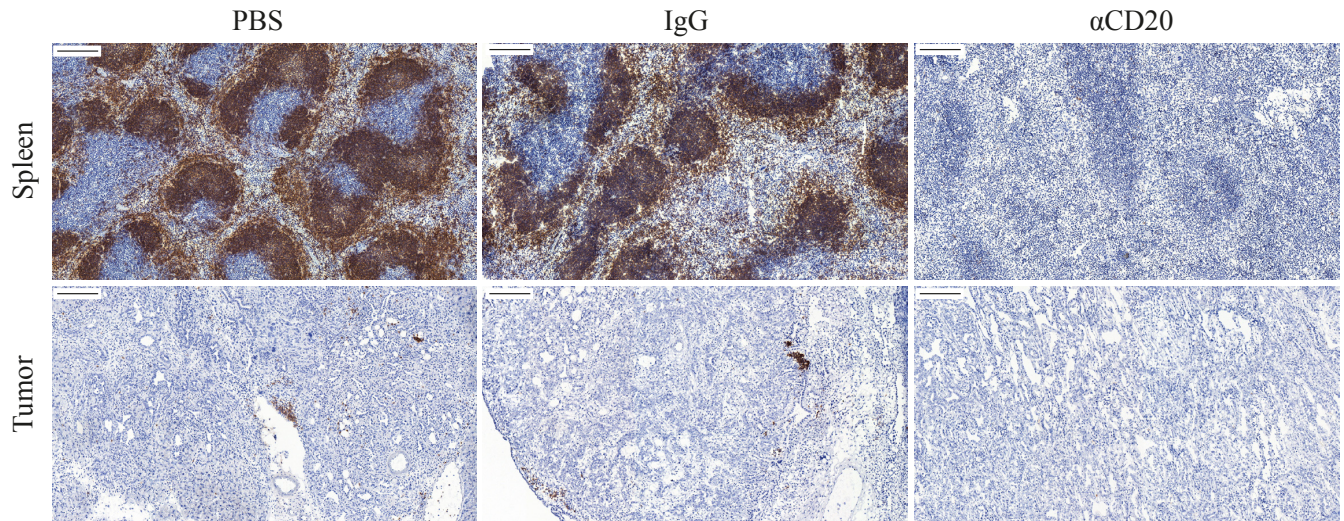

Supplement: Supplementary Figure S4 — The effect of B cell depletion in orthotopic PDAC. (A) Representative immunofluorescence images of absent immunoglobulin deposition of IgG1 (green channel) and IgG2a/b (white channel) near EpCAM positive tumor cells (red) where DAPI (blue) was used as a nuclear marker in μMT−/− tumors (n = 6). (B) Flow cytometry analysis of tumors of WT and μMT−/− mice. Upper panel from left to right, percentage of CD86+ TAMs (CD45+ CD11b+ Ly6G− Ly6C− F4/80+ MHC II+), CD86+ DCs (CD45+ CD11b+ Ly6G− Ly6C− F4/80− MHC II+ CD11c+), and CD206/Mannose receptor (MR)+ TAMs. Middle panel: ex vivo characterization of T cells from tumors of WT and μMT−/− mice following stimulation: from left to right, Ki67+ proliferation, IFNɤ+ and TNFa+ in CD8+ T cells (upper panels) and CD4+ T cells (lower panels), with the additional analysis of FOXP3+ Tregs. Each data point represents an individual mouse, mean and SD are also indicated. Statistical significance was tested using an unpaired t-test. (C) Representative IHC images of B220+ B cells (brown) on spleen and tumor sections from mice harboring orthotopic tumors treated with PBS (n = 7), IgG (n = 6), or anti-CD20 (n = 7), injected i.v. at −8, −2 pre and 14 days post-orthotopic surgery. Mice were culled at endpoint at 26–27 days. The scale bar represents 200 μm. [file Image_4.pdf]
