# Supplementary material for: Discrepancies in the Tumor Microenvironment of Spontaneous and Orthotopic Murine Models of Pancreatic Cancer Uncover a New Immunostimulatory Phenotype for B Cells
Source: Front Immunol. 2019 Mar 27;10:542. doi: 10.3389/fimmu.2019.00542 (PMC6445859; doi:10.3389/fimmu.2019.00542)

**Supplementary Figure 5 A-C**

A

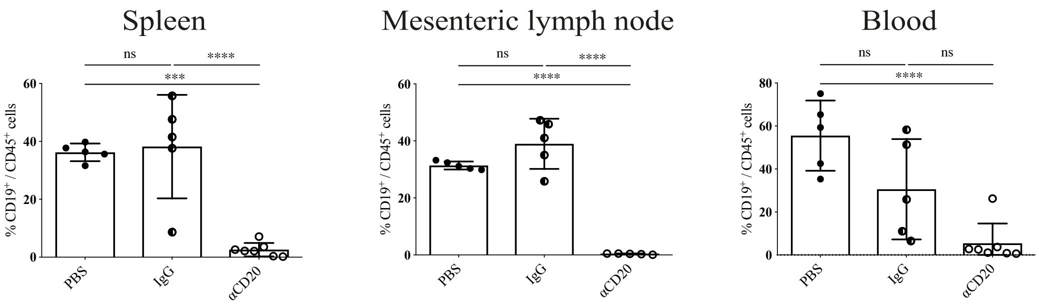


B Spleen

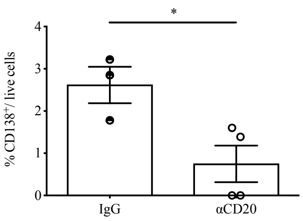


C Immunoglobulin deposition in tumors

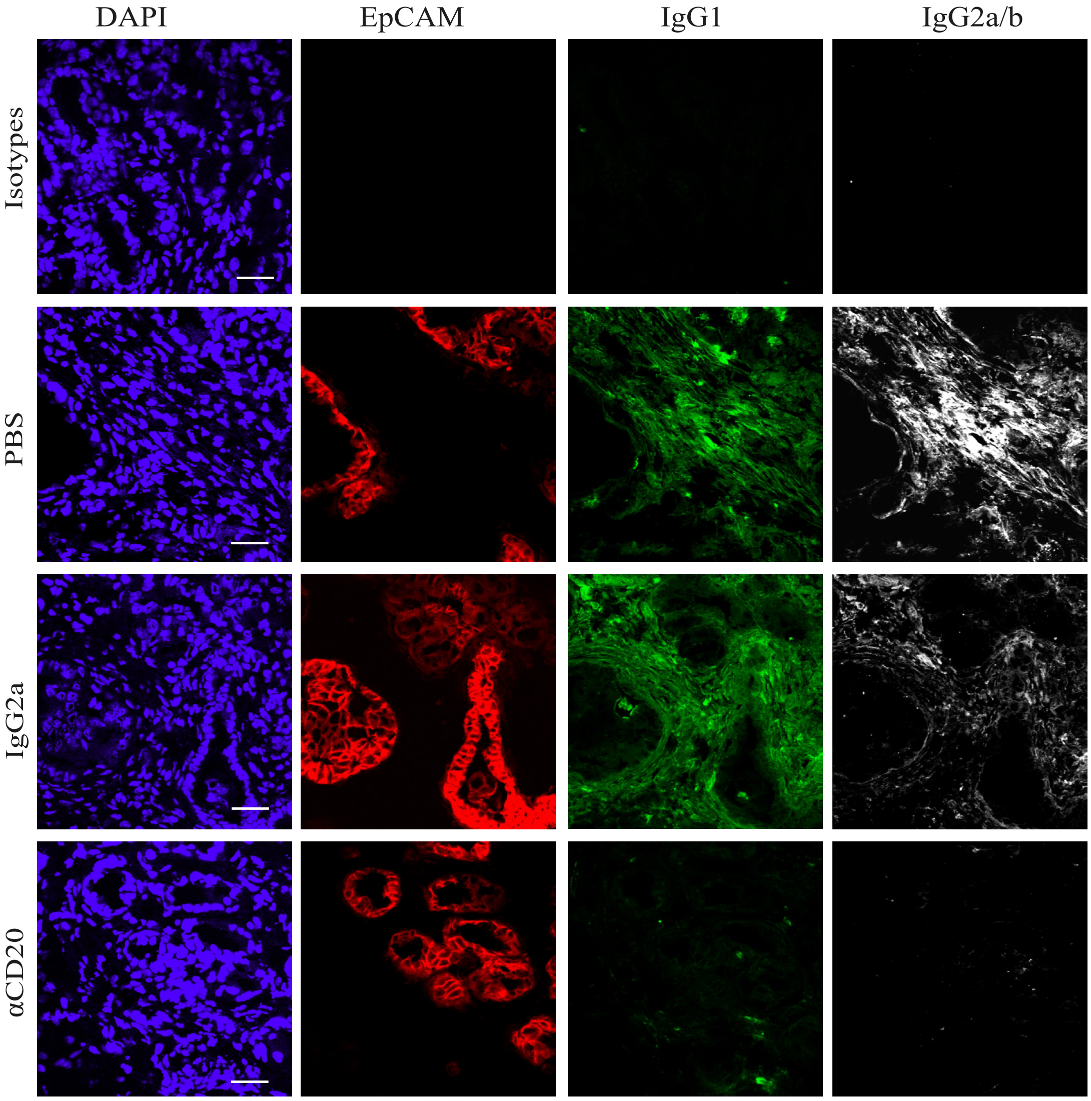

## Supplementary Figure 5 D-G

D

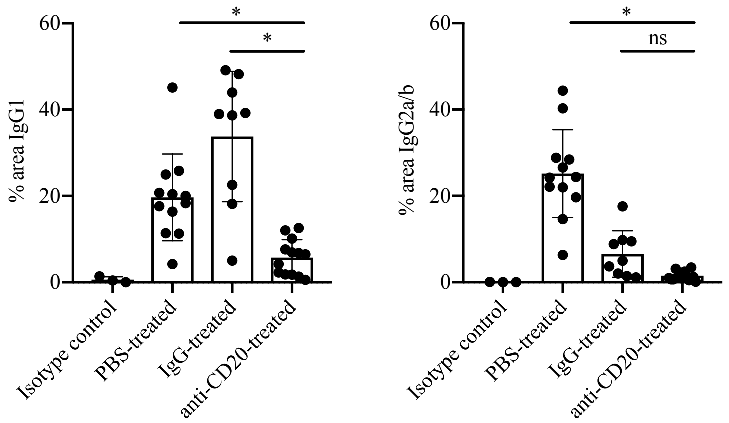


E

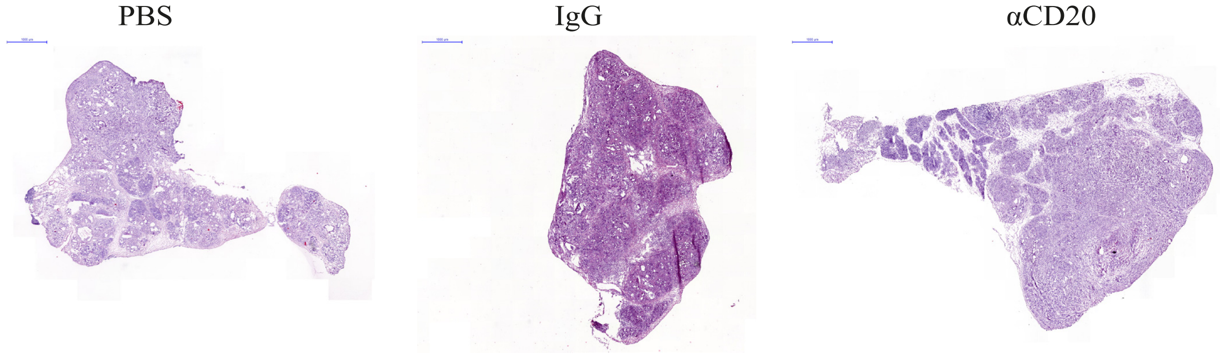


F

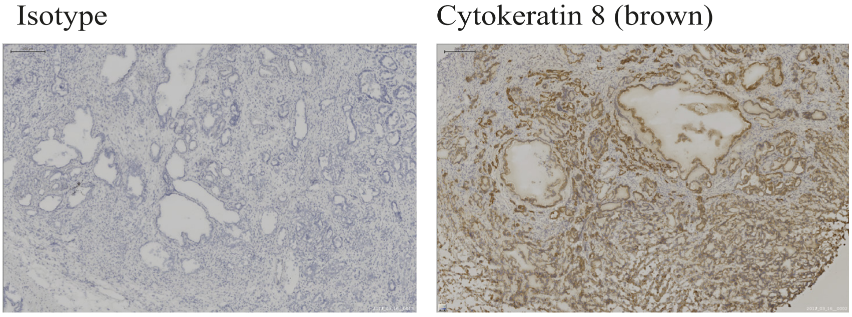


G

Tumor infiltrate flow cytometry analysis

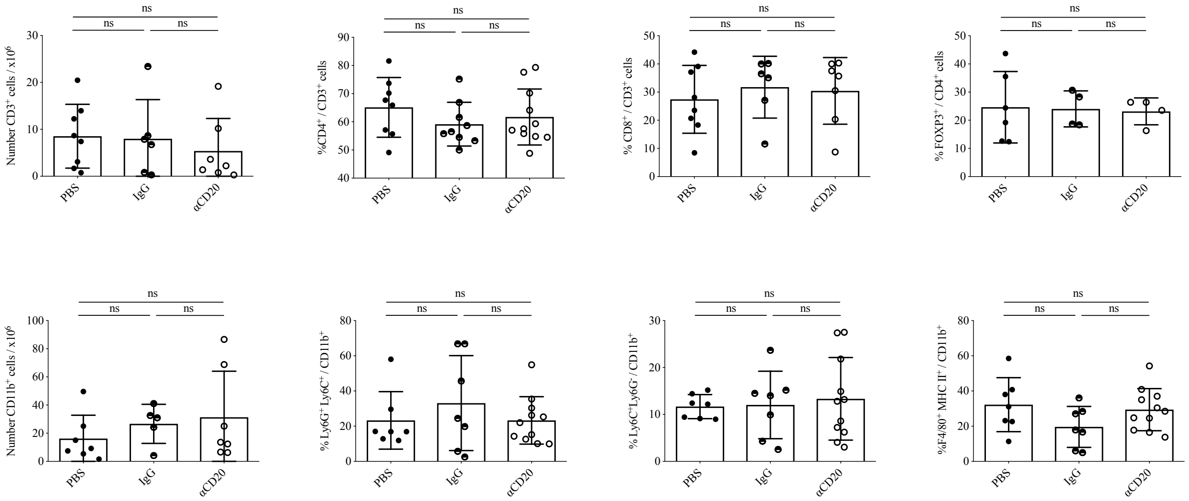

Supplement: Supplementary Figure S5 — Characterization of the effects of anti-CD20 B-cell depletion in KPC mice. (A) Flow cytometry analysis of the percentage of CD19+ cells out of CD45+ cells in the spleen, mesenteric lymph node and blood of KPC mice treated with PBS (n = 5), IgG2a (n = 5) or anti-CD20 (n = 5–7). (B) Flow cytometry of CD138hi plasma cells out of total live cells in the spleen of KPC mice treated with anti-CD20 (n = 4) compared to IgG2a-treated controls (n = 3). (C) Representative immunofluorescence images of immunoglobulin deposition of IgG1 (green) and IgG2a/b (white) near EpCAM positive epithelial/ tumor cells (red) where DAPI (blue) was used as a nuclear marker in KPC tumors following treatment with PBS, IgG2a of anti-CD20. Scale bar represents 50 μm. (D) Quantification of the percentage area of IgG1 and IgG2a/b (or respective isotype controls) on individual images of KPC tumor sections from mice that had been treated with either PBS, IgG2a or anti-CD20. Significance was tested using a One-Way ANOVA and post-hoc Tukey's multiple comparison test. (E) Representative hematoxylin stained sections of KPC tumors following treatment with PBS, IgG2a and anti-CD20, which were used to stage PanINs and percentage of PDAC per section. Scale bar is 1,000 μm. (F) Representative image of CK8 epithelial staining of KPC tumors from PBS- (n = 6), IgG- (n = 6), and anti-CD20-treated (n = 8) mice compared to isotype control. Scale bar is 200 μm. (G) Flow-cytometry analysis of immune cells isolated from tumors of PBS- (n = 6–8), IgG- (n = 4–9) and anti-CD20-treated (n = 4–11) mice. Graphs on upper panels from left to right show absolute number of CD3+ T cells, percentage of CD4+ cells out of CD3+ cells, percentage of CD8+ cells out of CD3+ cells, percentage of FOXP3+ (Tregs) out of CD3+CD4+. Graphs on lower panel from left to right show absolute number of CD11b+ (myeloid) cells, percentage of Ly6G+Ly6C+ (MDSCs) cells out of CD11b+ cells, percentage of Ly6G−Ly6C+ cells (monocytes) out of CD11b+ cells, pe [file Image_5.pdf]
