# Supplementary material for: Discrepancies in the Tumor Microenvironment of Spontaneous and Orthotopic Murine Models of Pancreatic Cancer Uncover a New Immunostimulatory Phenotype for B Cells
Source: Front Immunol. 2019 Mar 27;10:542. doi: 10.3389/fimmu.2019.00542 (PMC6445859; doi:10.3389/fimmu.2019.00542)
Supplement: Supplementary Table S1 — Antibodies used for flow cytometry. [file Table_1.pdf]

Supplementary Tables

Supplementary Table S1: Antibodies used for flow cytometry

Antibody	Fluorochrome	Clone	Company	Catalogue number
CD107a	PE	1D4B	Biolegend	121612
CD11b	Alexa Fluor 700	M1/70	eBioscience	56-0112
CD11b	APC	M1/70	eBioscience	17-0112-83
CD11b	Brilliant Violet 650	M1/70	Biolegend	101239
CD11c	Brilliant Violet 650	N418	Biolegend	117339
CD138	PE	281-2	Biolegend	142504
CD138	PE	281-2	BD	553714
CD19	Alexa Fluor 647	6D5	Biolegend	115522
CD19	FITC	eBIO1D3	eBioscience	11-0193-82
CD19	PerCP-Cy5.5	6D5	Biolegend	115534
CD19	PerCP-Cy5.5	eBio1D3	eBioscience	45-0193
CD19	PE/Cy5	eBio1D3	eBioscience	15-0193-83
CD206/ MR	PE	C068C2	Biolegend	141706
CD206/ MR	PE/Cy7	C068C2	Biolegend	141719
CD206/ MR	APC	C068C2	Biolegend	141708
CD3	PerCP-eFluor 710	17A2	eBioscence	46-0032-82

CD3	APC	17A2	Biolegend	100236
CD38	PerCP-eFluor 710	90	eBioscience	46-0381-80
CD4	FITC	GK1.5	eBioscience	11-0041
CD4	PerCP-Cy5.5	RM4-5	eBioscience	45-0042
CD4	PerCP-Cy5.5	RM4-5	Biolegend	100540
CD45	Brilliant Violet 605	30-F11	Biolegend	103140
CD45R/B 220	PE	RA3-6B2	eBioscience	12-0452
CD45R/B 220	PerCP-Cy5.5	RA3-6B2	eBioscience	45-0452
CD64/ FcγRI	PE	X54-5/7.1	Biolegend	139304
CD86	PerCP/Cy5.5	GL-1	Biolegend	105027
CD8a	Brilliant Violet 421	53-6.7	Biolegend	100738
F4/80	PE/Cy7	BM8	Biolegend	123114
F4/80	Brilliant Violet 650	BM8	Biolegend	123149
F4/80	Brilliant Violet 421	BM8	Biolegend	123137
FOXP3	APC	FJK-16s	eBioscience	17-5773-82
GL7	FITC	GL7	Biolegend	144604
IFN-γ	PE/Cy7	XMG1.2	Biolegend	505826
IgG1	FITC	X-56	Miltenyi	130-095-897

IgG2ab	APC	X-57	Miltenyi	130-095-875
IgG3	FITC	R40-82	BD Biosciences	553403
IgA	FITC	C10-3	BD Biosciences	559354
IL-10	PE	JES5-16E3	eBioscience	12-7101-82
Ki67	PE/Cy7	SolA15	eBioscience	25-5698
Ly6C	e-Fluor 450	HK1.4	eBioscience	48-5932
Ly6C	Brilliant Violet 785	HK1.4	Biolegend	128041
Ly6G	FITC	RB6-8C5	eBioscience	11-5931
Ly6G	PerCP-Cy5.5	1A8	Biolegend	127616
MHC II (I-A/I-E)	APC/Cy7	M5/114.15.2	Biolegend	107628
TNF-α	Alexa Fluor 647	MP6-X	Biolegend	506314
