# Supplementary material for: Discrepancies in the Tumor Microenvironment of Spontaneous and Orthotopic Murine Models of Pancreatic Cancer Uncover a New Immunostimulatory Phenotype for B Cells
Source: Front Immunol. 2019 Mar 27;10:542. doi: 10.3389/fimmu.2019.00542 (PMC6445859; doi:10.3389/fimmu.2019.00542)
Supplement: Supplementary Table S2 — Antibodies used in immunofluorescence and immunohistochemistry. [file Table_2.pdf]

Supplementary Table S2: Antibodies used in immunofluorescence and immunohistochemistry

Species raised in	Antibody	Fluoro-chrome	Clone	Company	Catalogue Number
Rat	B220	Purified	RA3-6B2	BD Biosciences	550286
Mouse	FcγRI	PE	X54-5/7.1	Biolegend	139304
Rabbit	CK8	Purified	Polyclona l	Abcam	59400
Rat	E-Cadherin	eFluor 660	DECMA-1	eBioscience	50-3249
Rat	EpCAM	PE	G8.8	eBioscience	12-5791
Rat	F4/80	Alexa Fluor 488	BM8	eBioscience	53-4801-82
Rat	F4/80	Purified	Cl: A3-1	AbD Serotec	MCA497
Rat	IgG1	FITC	X-56	Miltenyi	130-095-897
Rat	IgG2ab	APC	X-57	Miltenyi	130-095-875
Rat	IgG3	FITC	R40-82	BD Biosciences	553403
Rat	IgM	APC	II/41	eBioscience	17-5790-80
