# Supplementary material for: Discrepancies in the Tumor Microenvironment of Spontaneous and Orthotopic Murine Models of Pancreatic Cancer Uncover a New Immunostimulatory Phenotype for B Cells
Source: Front Immunol. 2019 Mar 27;10:542. doi: 10.3389/fimmu.2019.00542 (PMC6445859; doi:10.3389/fimmu.2019.00542)
Supplement: Supplementary Table S3 — Isotype antibody controls used in immunofluorescence and immunohistochemistry. [file Table_3.pdf]

Supplementary Table S3: Isotype antibody controls used in immunofluorescence and immunohistochemistry

Species	Isotype	Fluoro-chrome	Clone	Company	Catalogue Number
Rat	IgG1κ	APC	A110-1	BD Biosciences	550884
Rat	IgG1λ	APC	R3-34	BD Biosciences	554686
Rat	IgG1κ	FITC	eBRG1	eBioscience	11-4301-81
Mouse	IgG1κ	PE	MOPC-21	BD Biosciences	555749
Rat	IgG2a	Alexa Fluor488	54447	R&D	IC006G
Rat	IgG2aκ	FITC	eBR2a	eBioscience	11-4321
Rat	IgG2aκ	Purified	RTK2758	Biolegend	400501
Rat	IgG2aκ	PE	eBR2a	eBioscience	12-4321
Rabbit	IgG	Purified	Polyclonal	Cell Signaling	3900S
