# Supplementary material for: Discrepancies in the Tumor Microenvironment of Spontaneous and Orthotopic Murine Models of Pancreatic Cancer Uncover a New Immunostimulatory Phenotype for B Cells
Source: Front Immunol. 2019 Mar 27;10:542. doi: 10.3389/fimmu.2019.00542 (PMC6445859; doi:10.3389/fimmu.2019.00542)
Supplement: Supplementary Table S4 — Secondary antibodies used in immunohistochemistry. [file Table_4.pdf]

Supplementary Table S4: Secondary antibodies used in immunohistochemistry

Species raised in	Reactivity	Fluorochrome	Company	Catalogue Number
Rabbit	Rat IgG	Biotinylated	Vector Labs	BA-4001
Goat	Rabbit IgG	Biotinylated	Vector Labs	BA-1000
